# Mapping the distribution of neurotransmitters to resting-state functional connectivity in Parkinson’s disease

**DOI:** 10.1093/braincomms/fcaf308

**Published:** 2025-09-09

**Authors:** Weihua Li, Nicholas P Lao-Kaim, Runtian Li, Antonio Martín-Bastida, Andreas-Antonios Roussakis, Graham E Searle, Natalie Valle-Guzman, Viswas Dayal, Dilan Athauda, Zinovia Kefalopoulou, Philipp Mahlknecht, Alistair Church, Kathryn J Peall, Håkan Widner, Gesine Paul, Tom Foltynie, Roger A Barker, Paola Piccini

**Affiliations:** Division of Neurology, Department of Brain Science, Imperial College London, London W12 0NN, UK; Division of Neurology, Department of Brain Science, Imperial College London, London W12 0NN, UK; School of Artificial Intelligence, Beijing University of Posts and Telecommunications, Beijing 100876, China; Department of Neurology and Neurosciences, Clínica Universidad de Navarra, Pamplona-Madrid 31008, Spain; Division of National Heart & Lung Institute, Department of Vascular Science, Imperial College London, London SW3 6LY, UK; Invicro, London, W12 0NN, UK; Department of Public Health and Primary Care, University of Cambridge, Cambridge CB2 0SR, UK; Department of Clinical and Movement Neurosciences, University College London Institute of Neurology, London WC1N 3BG, UK; Department of Clinical and Movement Neurosciences, University College London Institute of Neurology, London WC1N 3BG, UK; Department of Clinical and Movement Neurosciences, University College London Institute of Neurology, London WC1N 3BG, UK; Department of Neurology, Innsbruck Medical University, 6020 Innsbruck, Austria; Royal Gwent Hospital, Cardiff, NP20 2UB, UK; Neuroscience and Mental Health Research Institute, Division of Psychological Medicine and Clinical Neurosciences, Cardiff University, Cardiff CF24 4HQ, UK; Division of Neurology, Department of Clinical Sciences, Lund University, Skåne University Hospital, Lund 22185, Sweden; Division of Neurology, Department of Clinical Sciences, Lund University, Skåne University Hospital, Lund 22185, Sweden; Translational Neurology Group, Department of Clinical Sciences, Wallenberg Neuroscience Centre, Lund University, Lund 22362, Sweden; Department of Clinical and Movement Neurosciences, University College London Institute of Neurology, London WC1N 3BG, UK; Department of Clinical Neuroscience, University of Cambridge, Cambridge CB2 0SZ, UK; Cambridge Stem Cell Institute, University of Cambridge, Cambridge CB2 0AW, UK; Division of Neurology, Department of Brain Science, Imperial College London, London W12 0NN, UK

**Keywords:** Parkinson’s disease, dopamine, serotonin, functional connectivity, PET imaging

## Abstract

Dopamine and serotonin are two major monoamine neurotransmitters associated with Parkinson's disease (PD), but their spatial distribution and relationship to underlying functional brain architecture are not fully understood. We assessed 30 patients with PD at baseline using structural MRI, resting-state functional MRI (rs-fMRI), ^11^C-PE2I and ^11^C-DASB PET, along with comprehensive clinical evaluations of motor and non-motor symptoms. Of these, 15 patients with PD who completed the same assessments after 19 months were included in the longitudinal analysis. rs-fMRI was used to assess functional connectivity, while ^11^C-PE2I and ^11^C-DASB PET were used to evaluate interregional homogeneity of dopamine and serotonin levels, referred to as PET covariance. Functional connectivity and PET covariance were estimated using a region-of-interest (ROI)-based approach with 138 ROIs from the Automated Anatomical Labelling 3 atlas, excluding cerebellar regions. These ROIs were further grouped into eight networks: visual, sensorimotor, attention, limbic, frontoparietal, default mode, subcortical and brainstem. At baseline, linear regression revealed that functional connectivity was positively associated with both ^11^C-PE2I PET covariance (*β*-values ranging from 0.575 to 0.790, *P* < 0.001) and ^11^C-DASB PET covariance (*β*-values ranging from 0.356 to 0.773, *P* < 0.001) across all networks. Longitudinally, we found positive correlations between baseline functional connectivity and both ^11^C-PE2I PET change covariance and ^11^C-DASB PET change covariance (*β*-values ranging from 0.166 to 0.576 and 0.312 to 0.671, respectively, *P* < 0.001) across all networks. These correlations remained significant after controlling for the Euclidean distance between ROIs, indicating that the association is independent of spatial proximity. For both tracers, absolute PET uptake across seed ROIs was positively associated with correspondent regression-derived functional connectivity-PET *β*-weights, which represent the relationship between PET uptake in target ROIs and their functional connectivity to the seed. This association between target functional connectivity and PET uptake was correlated with PD motor and non-motor severity across different brain regions in a manner that was dependent on the neurotransmitter system evaluated. Our findings suggest that in patients with PD, dopamine and serotonin levels covary among brain regions that are highly functionally connected. This implies that the spatial distribution of these neurotransmitters follows the organizational principles of the brain's functional connectomes, which are associated with features of the disease.

## Teaser

Li *et al*. report that dopamine and serotonin levels covary across functionally connected brain regions in Parkinson's disease. Their findings suggest that the spatial distribution of monoamine neurotransmitters aligns with the organizational principles of the brain's functional connectome, which are associated with features of the disease.

## Graphical Abstract

**Graphical Abstract:**
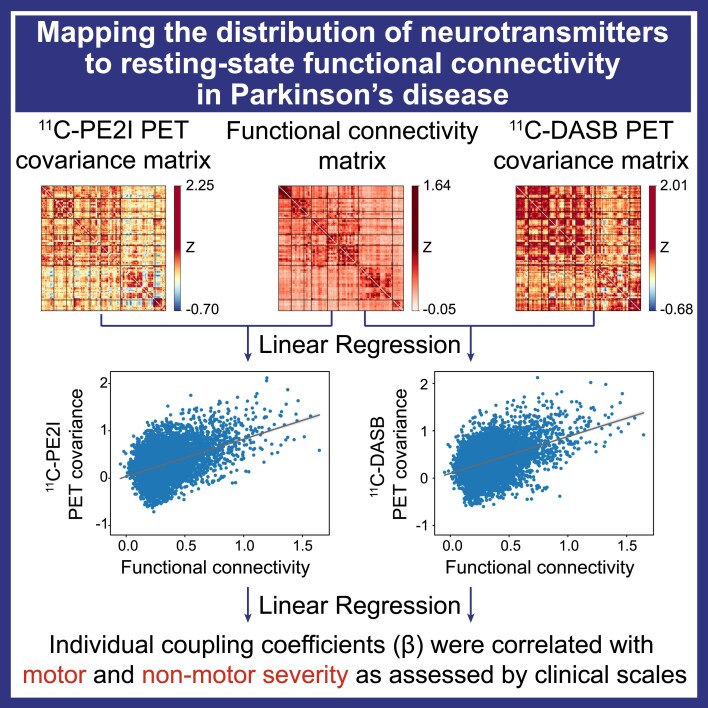

## Introduction

Dopamine and serotonin are two major monoamine neurotransmitters that are involved in motor control and the regulation of cognitive processes such as emotion, arousal and memory.^1-3^ Parkinson's disease (PD) is primarily characterized by the loss of dopaminergic neurons in the substantia nigra pars compacta.^4^ The resultant dopamine deficiency leads to the development of the classical Parkinsonian motor features, including resting tremor, bradykinesia and rigidity.^5^ However, the pathology of PD is not limited to the dopaminergic system, and an increasing body of evidence suggests that the serotonergic system is also affected in PD. The loss of serotonergic neurons may contribute to the development of non-motor symptoms such as depression, anxiety, cognitive impairment and sleep disorders,^6-8^ some of which can even precede motor symptoms.^9,10^

The positron emission tomography (PET) radioligands, ^11^C-PE2I and ^11^C-DASB, which are selective for the dopamine transporter (DAT) and serotonin transporter (SERT), respectively, have been used for assessing the integrity of the presynaptic dopaminergic and serotonergic systems *in vivo*.^11,12^ Previous studies have shown that there are alterations in both DAT and SERT density in the brains of patients with PD compared to healthy controls (HCs).^13-15^ Our previous ^11^C-PE2I PET study in patients with PD reported that progressive loss of dopaminergic neurons was correlated with the severity of bradykinesia and rigidity.^16^ In addition, several studies have shown altered prefrontal dopamine signalling in patients with PD,^17-19^ and these alterations could impair executive function.^20^  ^11^C-DASB PET studies showed reduced serotonergic function in the basal ganglia, which was associated with tremor severity during posture and action, but not with resting tremor.^21,22^ Another ^11^C-DASB PET study in patients with PD reported an overall increase in SERT binding in the dorsolateral and prefrontal cortices compared to HCs, with increased SERT binding in the orbitofrontal cortex being correlated with the severity of depression symptoms.^23^

Resting-state functional magnetic resonance imaging (rs-fMRI) can non-invasively assess the functional connectivity (FC) of neuronal activity by measuring synchronicity in the blood oxygen level-dependent (BOLD) signal, i.e. a proxy for neuronal firing.^24^ Evidence suggests that neurotransmitters, such as dopamine and serotonin, can influence resting-state FC by modulating the activity of neural circuits.^11,25^ On the other hand, altered resting-state FC may impact on neurotransmitter activity, which involves the release, receptor binding and reuptake of neurotransmitters.^26^ An earlier study in HCs demonstrated a greater degree of neurotransmitter receptor similarity between pairs of functionally connected brain regions, suggesting that these regions are likely to be co-modulated.^27^ However, it remains unclear how resting-state FC co-fluctuates with interregional patterns of dopamine or serotonin activity in PD, and whether this predicts future decline rates among those regions. Investigating how resting-state FC correlates with the spatial distribution and decline of these neurotransmitters will improve our understanding of the neural activity-associated pathological mechanisms underlying PD. This may explain the patterns of neurodegeneration observed in PD, thereby promoting the development of more effective treatments.^28,29^

The primary aim of this study was to examine whether higher FC between brain regions is associated with greater interregional homogeneity of dopamine and serotonin levels. To quantify this homogeneity, we correlated the uptake of ^11^C-PE2I and ^11^C-DASB PET between pairs of brain regions in 30 patients with PD. We refer to these measurements as ^11^C-PE2I PET covariance and ^11^C-DASB PET covariance, respectively. We first assessed the association between FC (rs-fMRI) and spatial PET covariance in patients with PD. We hypothesized that higher FC would be associated with higher PET covariance between brain regions. Secondly, we assessed longitudinal ^11^C-PE2I and ^11^C-DASB PET data from 15 patients with PD, combining these data with the baseline rs-fMRI data, and further hypothesized that highly functionally connected brain regions would show similar rates of dopamine and serotonin decline. Lastly, we identified brain regions where these FC–PET coefficients were correlated with PD symptoms and severity.

## Materials and methods

### Participants

Patients with PD were recruited from the FP7 EC-funded TRANSEURO programme cohort, an open-label, multicentre clinical trial investigating the efficacy and safety of foetal dopaminergic cell grafts in patients with PD.^30^ All participants were diagnosed with idiopathic PD in accordance with the Queen Square Brain Bank criteria.^31^ A total of 30 patients with PD were included at baseline based on the availability of structural MRI, rs-fMRI, ^11^C-PE2I and ^11^C-DASB PET and comprehensive clinical evaluations covering both motor and non-motor symptoms. Of these, 15 patients with PD who also completed follow-up assessments after 19 months, including repeat structural MRI, ^11^C-PE2I PET, ^11^C-DASB PET scans and clinical evaluations, were included in the longitudinal analysis. Although rs-fMRI was also acquired at follow-up, only the baseline rs-fMRI data were used in this study, in accordance with the study design. None of the patients had undergone transplantation surgery. Additionally, none of the participants had a history of depression, scored <26 on the Mini-Mental State Examination (MMSE), had atypical or secondary Parkinsonism or were ineligible for MRI and PET scanning. Motor severity of patients with PD was assessed using the motor component of the Movement Disorder Society-Sponsored Revision of the Unified Parkinson's Disease Rating Scale (MDS-UPDRS-III)^32^ and the modified Hoehn and Yahr (H&Y) rating scale^33^ while patients were in the ‘OFF’ medication state. The non-motor severity of patients with PD was assessed using the non-motor component of the MDS-UPDRS (MDS-UPDRD-IA and B), Beck's Depression Inventory (BDI), Non-Motor Symptoms Scale (NMSS), Apathy Evaluation Scale (AES), Addenbrooke's Cognitive Examination Revised (ACER), MMSE and Parkinson's disease sleep scale (PDSS). All participants provided written informed consent in accordance with the Declaration of Helsinki. All aspects of the study were approved by the Health Research Authority, the NRES Research Ethics Committees of the UK (REC 12/EE/0096 and 10/H0805/73) and the UK Administration of Radioactive Substances Advisory Committee (ARSAC).

### Image acquisition

All patients with PD underwent MRI and PET scans at Invicro LLC, London, UK, after withdrawal of dopaminergic medication for at least 24 h for standard release and 48 h for prolonged-release preparations.^34^ The average interval between the PET and MRI scans was 1–2 days.

MRI scans were conducted on a 3T Siemens Magnetom Trio scanner equipped with a 32-channel head coil. A three-dimensional T1-weighted sagittal magnetization-prepared rapid gradient-echo sequence was used to acquire structural MRI. The parameters for the sequence were as follows: repetition time (TR) of 2300 ms; echo time (TE) of 2.98 ms; flip angle of 9°; bandwidth of 240 Hz/Px; GRAPPA acceleration factor of 2; slice thickness of 1 mm (without any gap); field of view (FOV) of 240 × 256 mm; and matrix size of 240 × 256. Each structural MRI scan lasted 5 min, and a whole-brain volume consisting of 160 slices was obtained. For rs-fMRI, a T2*-weighted single-shot gradient-echo echo planar imaging sequence was employed to acquire a total of 144 brain volumes for each participant. The imaging parameters used were as follows: TR of 2500 ms; TE of 31.3 ms; flip angle of 80°; bandwidth of 2298 Hz/Px; GRAPPA acceleration factor of 2; slice thickness of 3 mm (without any gap); FOV of 192 × 192 mm and matrix size of 64 × 64.

A Siemens Biograph TruePoint HI-REZ PET/computed tomography (CT) system was utilized to perform ^11^C-PE2I and ^11^C-DASB PET scans. A low-dose CT transmission scan (0.36 mSv) was conducted to adjust for attenuation. Radioligand volumes (^11^C-PE2I = 350 MBq; ^11^C-DASB = 450 Mbq) were prepared to 10 ml using saline solution and administered intravenously as single bolus injections, followed immediately by a 10 ml saline flush. The rate of administration was 1 ml per second. Dynamic emission data were collected for a duration of 90 min following the injection. These data were then processed using a filtered back-projection technique, namely the direct inversion Fourier transform. The reconstruction parameters were as follows: matrix size of 128 × 128; zoom factor of 2.6; transaxial Gaussian filter of 5 mm and pixel size of 2 mm (isotropic).

### Image preprocessing

All PET images were analysed using the Molecular Imaging and Kinetic Analysis Toolbox software package for academic use (MIAKAT™),^35^ which implements FSL (FMRIB Image Analysis Group, Oxford, UK),^36^ SPM12 (Statistical Parametric Mapping, Wellcome Trust Centre for Neuroimaging, London, UK, http://www.fil.ion.ucl.ac.uk/spm/) and in-house preprocessing and kinetic modelling procedures. Dynamic PET images were motion-corrected using frame-to-frame rigid registration to a pre-specified reference frame (frame = 16). Signal-averaged (summed) images were generated by adding frames that ranged from 10 to 90 min. These images were subsequently co-registered with the corresponding structural MRI. To minimize the impact of partial volume effects, partial volume correction was applied to all PET data using co-registered structural MRI, ensuring more accurate quantification of tracer binding. Regional time-activity curves were generated by registering the automated anatomical labelling 3 (AAL3) atlas to dynamic PET frames. The simplified reference tissue model^37^ using cerebellar grey matter as a reference region was employed to generate parametric ^11^C-PE2I and ^11^C-DASB non-displaceable binding potential (*BP_ND_*) images, reflecting DAT and SERT availability, respectively.

All rs-fMRI data were analysed using SPM12. The first 10 volumes were removed to avoid saturation effects and allow for magnetization equilibrium. The remaining volumes were slice-time corrected, realigned to the mean functional image and denoised by regressing out signal from white matter and cerebrospinal fluid, as well as the Friston 24 motion parameters (i.e. 6 motion parameters, 6 motion parameters one time point before and the 12 corresponding squared items).^38^ Next, detrending, band-pass filtering (0.01–0.08 Hz) and despiking with a threshold of 2.5 SD were applied. To further eliminate motion artefacts, the framewise displacement (FD) of every time point *t* was calculated, and high-motion volumes that exceeded a pre-defined limit (FD (*t*) > 0.5 mm) were removed. Lastly, the preprocessed rs-fMRI images were spatially normalized to the Montreal Neurological Institute (MNI) space using the DARTEL model and then smoothed with a 6-mm full width at half maximum Gaussian kernel to reduce spatial noise. It should be noted that we did not perform global signal regression due to certain disagreements regarding the possible bias imposed by this preprocessing step.^39^

### FC and PET covariance

FC and PET covariance were estimated in a region-of-interest (ROI)-based manner, using 138 ROIs from the AAL3 atlas, excluding cerebellar regions.^40^ The 138 ROIs were grouped into eight networks according to previous parcellations,^41,42^ which consists of the visual (VN), sensorimotor (SMN), attention (AN), limbic (LN), frontoparietal (FPN), default mode (DMN), subcortical (SUB) and brainstem (BN) networks (see Supplementary Table 1). This atlas is well-suited for our combined analysis of rs-fMRI, ^11^C-PE2I and ^11^C-DASB PET because it defines a number of regions that are strongly related to PD, such as the substantia nigra, raphe nucleus, caudate and putamen. Additionally, we calculated the Euclidean distance between ROIs, which is the geometric distance between the centre of each ROI, to subsequently determine if the distance between ROIs may account for associations between FC and PET uptake.

### FC assessment

FC was estimated for each participant based on fully preprocessed rs-fMRI data. Specifically, we extracted the rs-fMRI time course for each of the 138 ROIs by averaging the signal across voxels within an ROI per time point. These ROI time courses were then correlated using Pearson's correlation, resulting in a 138 × 138 FC matrix that was Fisher *z*-transformed, with autocorrelations set to zero. Lastly, we computed the group-average FC matrix for the patients with PD. To derive network-level FC, we calculated the average of all ROI-to-ROI correlation values among ROIs within each predefined network rather than averaging across all voxels.

### Assessing cross-sectional and longitudinal covariance of PET

Cross-sectionally, we assessed the correlation between the uptake of PET in a given region X and another region Y. We first extracted PET uptake from each of the 138 ROIs for each patient with PD. Next, we vectorized the ROI data into 138-element vectors. We then assessed the pairwise ROI-to-ROI Spearman's correlation of PET uptake across participants using these 138-element vectors. This procedure was used to generate a 138 × 138 PET covariance matrix for both ^11^C-PE2I and ^11^C-DASB at baseline. Longitudinal covariance matrices were derived using the change in PET uptake between visits (follow-up—baseline). To avoid possible issues with non-Gaussian distributions, we explicitly employed Spearman's correlations throughout our analyses. All correlations were Fisher *z*-transformed, making them equivalent to the FC matrices, with autocorrelations set to zero.

### Statistical analysis

The Shapiro–Wilk test was conducted to determine whether the data were normally distributed. Baseline demographic differences between male and female patients with PD were assessed using independent-samples *t*-tests for normally distributed data and the Mann–Whitney U-test for non-normally distributed data. Differences in demographics between baseline and follow-up within the PD subgroup were assessed using paired *t*-tests for normally distributed data and the Wilcoxon signed-rank tests for non-normally distributed data.

Lateralized UPDRS-III score at baseline was used to determine which sides were clinically most and least affected. Given that the caudate and putamen are considered to be among the most severely affected regions in PD, we performed a paired *t*-test to explore whether PET uptake in these regions differed between the clinically most and least affected (MA/LA) sides and to assess whether there was a significant longitudinal change in PET uptake. We considered results statistically significant at a False Discovery Rate (FDR)-corrected *P* < 0.05.

Cross-sectionally, we first performed linear regression with the vectorized group-average FC matrix as the independent variable and the vectorized PET covariance matrix as the dependent variable. We also assessed the association between FC and PET covariance for each of the eight brain networks separately. The Steiger's *Z* procedure was employed to evaluate whether the correlation coefficients describing the association between FC and PET covariance differed significantly between ^11^C-PE2I and ^11^C-DASB. Furthermore, we repeated the above analysis while controlling for Euclidean distance between ROIs in the regression model to determine whether the associations between FC and PET covariance were independent of spatial proximity between ROIs.

In the next step, we vectorized and rank-ordered all ROIs by group-average PET uptake. Then, we evaluated the group-average FC between each rank-ordered ROI (seed region) and the remaining ROIs (target regions). The PET uptakes in the target ROIs for a given seed were then regressed onto these FC measures. This procedure was repeated for all seed ROIs, yielding a sequence of *β*-values (see Supplementary Fig. 1). To examine whether PET uptake in any given seed ROI is associated with the FC–PET relationship of its targets, we calculated the Spearman's correlation between PET uptake across seed ROIs and their corresponding *β*-value. FC–PET associations for seed regions with the highest (hotspot) and lowest (coldspot) PET uptake [see Supplementary Fig. 1 (iii) and Supplementary Table 2] were plotted for comparison.

The same analysis methods were used in the longitudinal analysis, substituting the change in PET uptake between baseline and follow-up (i.e. follow-up—baseline) for the baseline PET uptake.

Finally, FC–PET association maps were generated for each patient with PD and each tracer. Linear regression was performed for each seed ROI, with the vectorized FC to the remaining 137 target ROIs as the independent variable and the corresponding vectorized PET uptake as the dependent variable, to obtain a 138-long *β*-vector representing the FC–PET association across the whole brain. Spearman's correlation was then used to identify brain regions where these FC–PET coefficients were correlated with PD symptoms and severity (see Supplementary Fig. 2 for an analysis schematic). In the longitudinal analysis, the same analysis methods were used. The change in PET uptake and clinical measures between baseline and follow-up (i.e. follow-up minus baseline) was substituted for the baseline PET uptake and clinical measures, respectively (see Supplementary Fig. 3 for an analysis schematic). We considered results statistically significant at *P* < 0.05 (FDR-corrected).

## Results

### Participant characteristics

Demographic and clinical characteristics of study participants are summarized in Table 1. As expected, patients with PD showed significantly higher levodopa equivalent doses at follow-up compared with baseline. Although some fluctuations in both motor and non-motor scales were observed, these were not significant.

**Table 1:** Demographic and clinical information for all PD participants at baseline (*PD: n = 30*) and for the PD subgroup at baseline and follow-up (*PD_FU_: n* = *15*)

*Baseline: PD (Male: n = 24; Female: n = 6)*				
	Male	Female	Statistic	*P*
Age (years)^a^	54.8 ± 7.2	57.6 ± 7.2	*t*(28) = −0.844	0.406
Disease duration (years)^a^	6.0 ± 2.3	5.1 ± 1.9	U = 94.000	0.273
UPDRS-III^a^	33.0 ± 10.5	24.2 ± 7.9	*t*(28) = 1.924	0.065
Bradykinesia-rigidity sub-score^a^	23.3 ± 8.2	18.8 ± 7.3	*t*(28) = 1.226	0.230
Tremor sub-score^a^	7.0 ± 5.5	2.7 ± 2.5	*t*(28) = 1.869	0.072
Hoehn and Yahr Scale^b^	2.0 (0.0)	2.0 (0.0)	U = 84.000	0.055
UPDRS-I A^a^	4.5 ± 3.1	6.5 ± 5.7	U = 35.000	0.470
UPDRS-I B^a^	5.6 ± 3.8	8.3 ± 9.2	U = 39.500	0.677
UPDRS-I^a^	5.8 ± 5.0	6.8 ± 3.3	U = 34.000	0.425
BDI^a^	4.2 ± 3.6	5.5 ± 5.0	*t*(26) = −0.609	0.548
NMSS^a^	17.3 ± 14.9	5.0 ± 4.6	U = 51.000	0.143
AES^a^	27.4 ± 7.2	21.7 ± 1.5	*t*(24) = 1.339	0.193
ACER^a^	98.5 ± 1.6	98.8 ± 0.5	U = 54.000	0.706
MMSE^a^	29.8 ± 0.6	29.8 ± 0.5	U = 51.000	0.806
PDSS^a^	114.5 ± 13.7	109.7 ± 46.3	U = 26.000	0.521
LED^a^	732.6 ± 365.4	421.0 ± 228.0	*t*(28) = 1.980	0.058

*Follow-up: PD_FU_ (Male: n* = *12; Female: n* = *3)*				
	Baseline	Follow-Up	Statistic	*P*
Age (years)^a^	53.0 ± 7.2	54.7 ± 7.1	*t*(14) = 26.445	<0.001***
Disease duration (years)^a^	6.0 ± 2.2	7.7 ± 2.0	*t*(14) = 26.234	<0.001***
UPDRS-III^a^	31.4 ± 12.0	35.9 ± 10.9	*t*(14) = 2.003	0.065
Bradykinesia-rigidity sub-score^a^	22.4 ± 9.6	25.2 ± 8.9	*t*(14) = −1.925	0.075
Tremor sub-score^a^	6.9 ± 4.5	8.3 ± 4.2	*t*(14) = −1.514	0.152
Hoehn and Yahr Scale^b^	2.0 (0.0)	2.0 (0.0)	*W* = 0.00	0.083
UPDRS-I A^a^	1.1 ± 1.8	0.8 ± 0.9	*W* = 13.000	0.478
UPDRS-I B^a^	4.2 ± 2.9	4.3 ± 3.3	*W* = 23.500	0.677
UPDRS-I^a^	5.5 ± 3.7	4.9 ± 3.6	*W* = 23.000	0.644
BDI^a^	5.3 ± 3.4	6.3 ± 5.2	*t*(12) = −1.237	0.240
NMSS^a^	13.5 ± 14.3	13.1 ± 9.7	*W* = 50.000	0.903
AES^a^	29.8 ± 7.7	29.3 ± 8.2	*t*(10) = 0.443	0.667
ACER^a^	98.4 ± 1.7	98.4 ± 1.0	*W* = 20.500	0.809
MMSE^a^	29.8 ± 0.6	29.7 ± 0.8	*W* = 4.500	0.854
PDSS^a^	115.1 ± 17.1	104.3 ± 23.5	*t*(10) = 1.386	0.196
LED^a^	771.7 ± 386.5	906.3 ± 235.7	*t*(14) = 2.156	0.046*

^a^Data are presented as mean ± SD; ^b^Data are presented as median (interquartile range); *t = *t-*test; U = Mann–Whitney U-test; *W* = Wilcoxon signed-rank test Indicates *P* < 0.05; ***Indicates *P* < 0.001; UPDRS = Unified Parkinson's disease rating scale; BDI = Beck's depression inventory; NMSS = Non-Motor Symptoms Scale; AES = Apathy Evaluation Scale; ACE-R = Addenbrooke's Cognitive Examination Revised; MMSE = Mini-mental State Examination; PDSS = Parkinson's disease sleep scale; LED = levodopa equivalent dose (mg); Clinical scales were assessed in the practically defined off-medication state.

### Group-average ^11^C-PE2I and ^11^C-DASB PET of patients with PD

**Figure 1:**
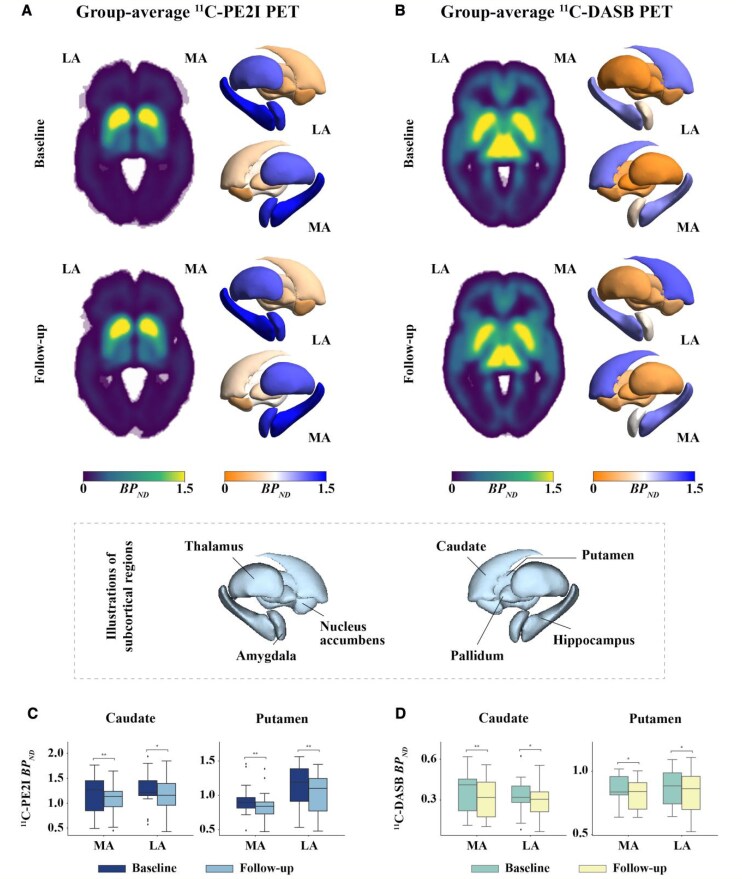
**Group-average parametric ^11^C-PE2I and ^11^C-DASB *BP_ND_* images in patients with PD at baseline (*N* = 30) and follow-up (*N* = 15).** (**A**) Group-average parametric ^11^C-PE2I and (**B**) ^11^C-DASB *BP_ND_* images derived from 15 patients with PD at baseline and follow-up. (**C**) Boxplots of caudate and putamen ^11^C-PE2I and (**D**) ^11^C-DASB *BP_ND_* for the same patients (*N* = 15) at baseline and follow-up. Striatal values were obtained from the clinically MA and LA sides separately. Each data point in the boxplots represents the *BP_ND_* value from an individual patient, for a given brain region and time point. Statistical comparisons were conducted using paired *t*-tests to assess differences between MA and LA sides, as well as longitudinal changes over time. The *P*-values were FDR-corrected to account for multiple comparisons. * indicates *P* < 0.05; ** indicates *P* < 0.01; *** indicates *P* < 0.001.

Figure 1A and B show group-average parametric ^11^C-PE2I and ^11^C-DASB non-displaceable binding potential (*BP_ND_*) images derived from 15 patients with PD who had both baseline and follow-up data. The boxplots of caudate and putamen ^11^C-PE2I *BP_ND_* and ^11^C-DASB *BP_ND_* at baseline and follow-up are shown in Fig. 1C and D, respectively. Striatal PET uptake was obtained from the clinically MA and LA sides separately. There was no significant difference in ^11^C-PE2I *BP_ND_* or ^11^C-DASB *BP_ND_* between the clinically MA and LA sides for either the putamen or caudate. However, ^11^C-PE2I *BP_ND_* and ^11^C-DASB *BP_ND_* were significantly decreased at follow-up compared with baseline. The *P*-values were FDR-corrected to account for multiple comparisons.

### FC is associated with PET covariance at baseline

**Figure 2:**
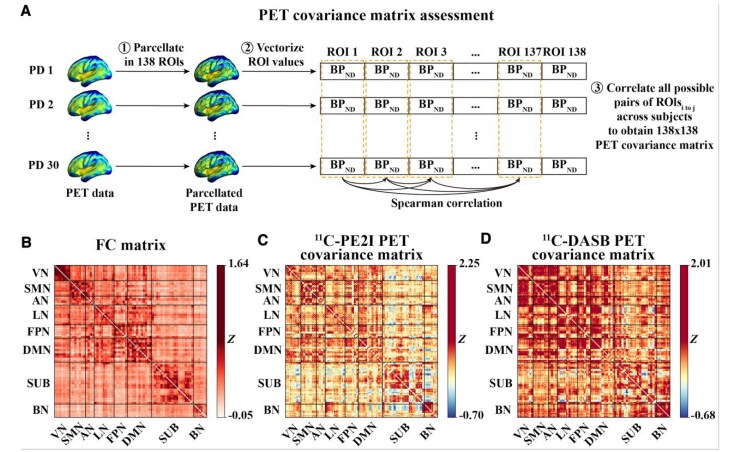
**Group-average FC and PET covariance matrices at baseline.** (**A**) Flow chart illustrating the analysis pipeline for PET covariance computation. (**B**) Group-average FC matrix across 30 patients with PD, computed using Pearson correlation and Fisher *z*-transformation. (**C**) Group-average ^11^C-PE2I PET covariance matrix and (**D**) group-average ^11^C-DASB PET covariance matrix, both computed using Spearman's correlation between pairwise ROI-to-ROI PET *BP_ND_* values. Abbreviations: VN = Visual Network; SMN = Sensorimotor Network; AN = Attention Network; LN = Limbic Network; FPN = Frontoparietal Network; DMN = Default Mode Network; SUB = Subcortical Network; BN = Brainstem Network.

The analysis pipeline for PET covariance computation is summarized in the flow chart presented in Fig. 2A. rs-fMRI data were used to obtain the group-average FC matrix for patients with PD (Fig. 2B). The ^11^C-PE2I and ^11^C-DASB PET covariance matrices are shown in Fig. 2C and D, respectively.

**Figure 3:**
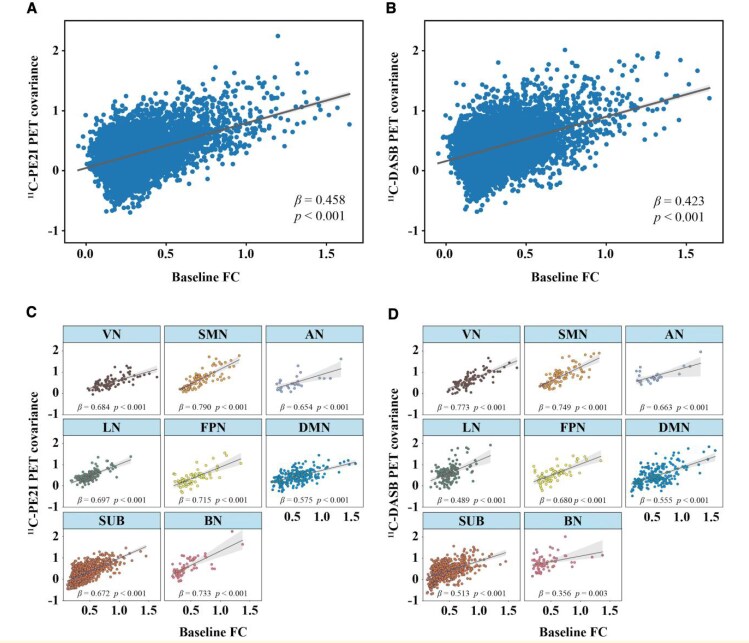
**Association between FC and PET covariance in patients with PD (*N* = 30) at baseline.** Scatterplots showing the association between group-average FC and (**A**, **C**) ^11^C-PE2I PET covariance and (**B**, **D**) ^11^C-DASB PET covariance for the whole brain and for each of the eight brain networks separately. Statistical analysis was performed using linear regression, with the vectorised group-average FC matrix as the independent variable and the vectorised PET covariance matrix as the dependent variable. Each data point in the scatterplots represents a pairwise comparison between two brain regions (ROIs), reflecting the strength of their FC and corresponding PET covariance. For whole-brain analyses, 9453 ROI pairs were included per modality. Network-level associations were also evaluated using the same model, with the number of ROI pairs per network ranging from 28 to 703, depending on the number of ROIs in each network (ROI pairs per network: VN: *n* = 91; SMN: *n* = 91; AN: *n* = 28; LN: *n* = 153; FPN: *n* = 66; DMN: *n* = 231; SUB: *n* = 703; BN: *n* = 66). All *P*-values were FDR-corrected to account for multiple comparisons. Abbreviations: VN = Visual Network; SMN = Sensorimotor Network; AN = Attention Network; LN = Limbic Network; FPN = Frontoparietal Network; DMN = Default Mode Network; SUB = Subcortical Network; BN = Brainstem Network.

**Figure 4:**
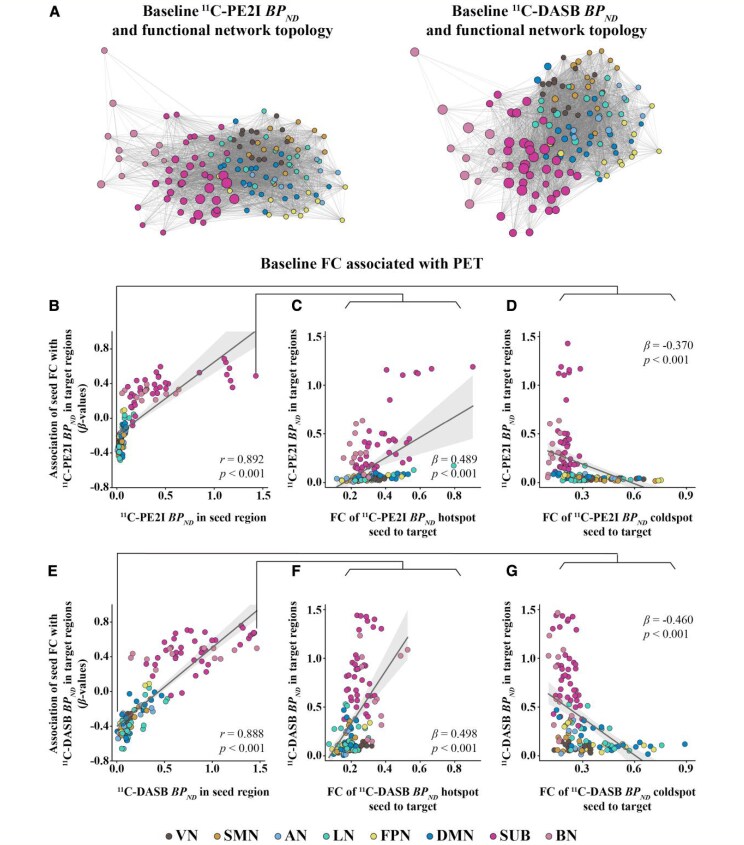
**FC as a predictor of PET uptake in patients with PD (*N* = 30).** (**A**) Force-directed graphs illustrating the association between functional network topology (node distance), and ^11^C-PE2I or ^11^C-DASB PET uptake (node size). (**B** and **E**) Scatterplots showing, for each of the 138 seed ROIs, the association between its own PET uptake (*x*-axis) and the corresponding *β*-value (*y*-axis), derived by regressing group-average FC from the seed to each of the 137 target ROIs onto PET uptake in the respective target regions. Illustration of the results shown for regions of highest (hotspot) and lowest (coldspot) ^11^C-PE2I (**C, D**) and ^11^C-DASB (**F**, **G**) PET uptake. For each seed region, group-average FC with all target ROIs was regressed onto the corresponding PET uptake in the targets, yielding a *β*-value. This was repeated for all 138 seed ROIs. To assess whether seed-level PET uptake is associated with the FC–PET relationship across the brain, Spearman's correlation was used to compare PET uptake in each seed ROI with its corresponding *β*-value. Each data point in figures represents one seed ROI (*n* = 138). All *P*-values were FDR-corrected to account for multiple comparisons.

Linear regression with the vectorized PD group matrices showed a positive relationship between FC and ^11^C-PE2I PET covariance (*β* = 0.458, *P* < 0.001, Fig. 3A) and between FC and ^11^C-DASB PET covariance (*β* = 0.423, *P* < 0.001, Fig. 3B). Here, the associations between FC and PET covariance remained significant when controlling for Euclidean distance between ROIs in the regression model (^11^C-PE2I PET: *β* = 0.334, *P* < 0.001; ^11^C-DASB PET: *β* = 0.328, *P* < 0.001). Furthermore, we found significant positive correlations between FC and ^11^C-PE2I PET covariance (*β*-values ranging from 0.575 to 0.790, *P* < 0.001, Fig. 3C) and between FC and ^11^C-DASB PET covariance (*β*-values ranging from 0.356 to 0.773, *P* < 0.01, Fig. 3D) within all eight brain networks. The *P*-values shown in the figure were FDR-corrected to account for multiple comparisons. Steiger's *Z* indicated that the correlation between FC and ^11^C-PE2I PET covariance for the whole brain (*Z* = 2.858, *P* = 0.004), SMN (*Z* = 8.473, *P* < 0.001), LN (*Z* = 21.221, *P* < 0.001), FPN (*Z* = 3.502, *P* < 0.001), SUB (*Z* = 16.069, *P* < 0.001) and BN (*Z* = 36.750, *P* < 0.001) networks was significantly stronger than the correlation between FC and ^11^C-DASB PET covariance, while in the VN (*Z* = −13.704, *P* < 0.001), the correlation was significantly weaker for ^11^C-PE2I PET covariance than for ^11^C-DASB PET covariance (see Supplementary Table 3). Projecting ^11^C-PE2I and ^11^C-DASB PET uptake onto the functional network topology showed that the degree of PET uptake tended to cluster within highly functionally connected regions (Fig. 4A).

### PET uptake correlates with FC–PET associations in target ROIs

We found a significant positive correlation between absolute PET uptake across seed ROIs and their correspondent regression-derived *β*-value, representing the relationship between PET uptake in the target ROIs and their FC to the seed ROI for both ^11^C-PE2I PET (*r* = 0.892, *P* < 0.001, Fig. 4B) and ^11^C-DASB PET (*r* = 0.888, *P* < 0.001, Fig. 4E). Specifically, for seeds with higher ^11^C-PE2I PET uptake, higher FC to target ROIs was associated with higher ^11^C-PE2I PET uptake in target regions. In contrast, for seeds with lower ^11^C-PE2I PET uptake, higher FC to target ROIs was associated with lower ^11^C-PE2I PET uptake in target regions. The same was found for ^11^C-DASB PET. To further illustrate this concept, we present the results of the analysis on seed ROIs with the highest (hotspot) and lowest (coldspot) PET uptake for both ^11^C-PE2I PET (Fig. 4C and D) and ^11^C-DASB PET (Fig. 4F and G). The *P*-values shown in the figure were FDR-corrected to account for multiple comparisons.

### FC is associated with PET change covariance

**Figure 5:**
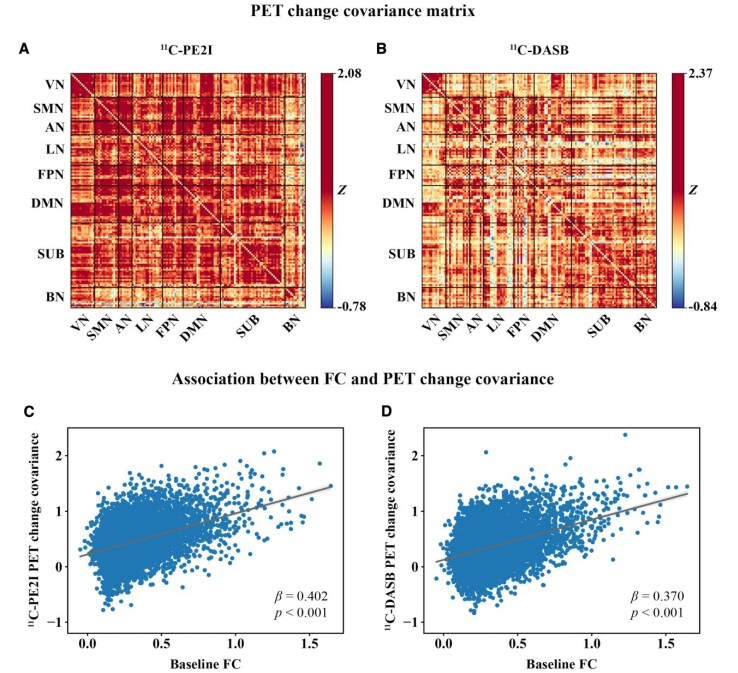
**Association between baseline FC and PET change covariance in patients with PD (*N* = 15).** (**A**) Group-average ^11^C-PE2I and (**B**) ^11^C-DASB PET change covariance matrices, calculated using Spearman's correlation between pairwise ROI-to-ROI PET *BP_ND_* change values (follow-up—baseline) across 15 patients with PD. Scatterplots showing the association between baseline group-average FC and (**C**) ^11^C-PE2I PET change covariance and (**D**) ^11^C-DASB PET change covariance for the whole brain. Statistical analysis was performed using linear regression, with vectorized baseline FC as the independent variable and vectorized PET change covariance as the dependent variable. Each data point in the scatterplots represents a pairwise comparison between two brain regions (ROIs), reflecting the strength of their FC and corresponding PET change covariance. For whole-brain analyses, 9453 ROI pairs were included per modality. This approach follows the same method used in the cross-sectional analysis, substituting PET change values for baseline uptake. All *P*-values were FDR-corrected to account for multiple comparisons. Abbreviations: VN = Visual Network; SMN = Sensorimotor Network; AN = Attention Network; LN = Limbic Network; FPN = Frontoparietal Network; DMN = Default Mode Network; SUB = Subcortical Network; BN = Brainstem Network.

**Figure 6:**
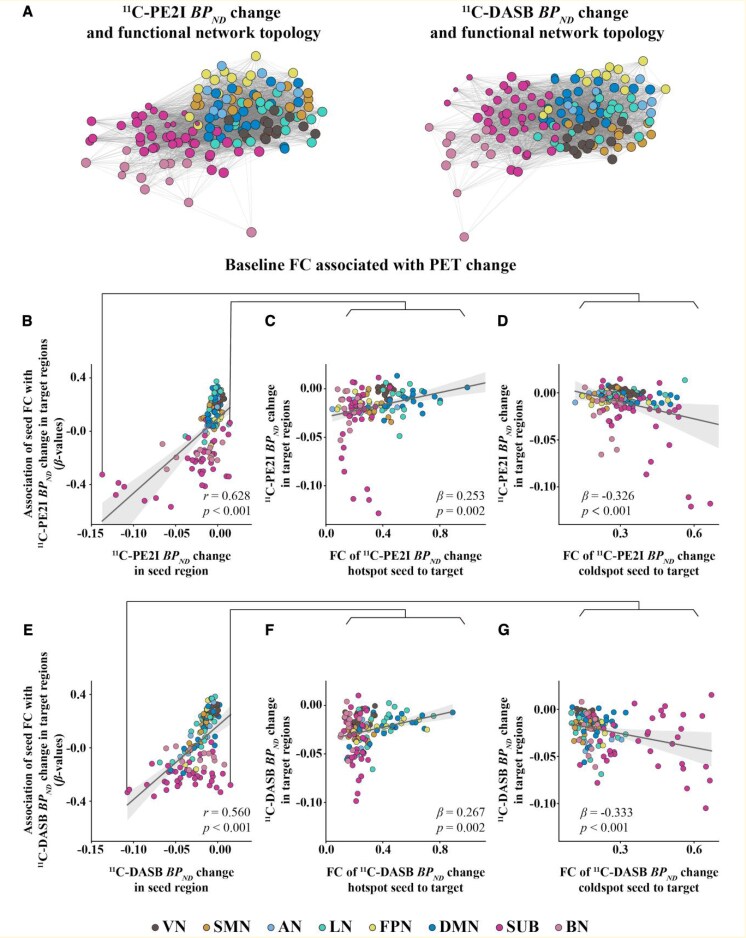
**FC as a predictor of longitudinal changes in PET uptake in patients with PD (*N* = 15).** (**A**) Force-directed graphs illustrating the association between functional network topology (node distance), and ^11^C-PE2I or ^11^C-DASB PET uptake changes (node size), calculated as the difference in ^11^C-PE2I and ^11^C-DASB PET uptake between follow-up and baseline. Scatterplots showing the association between (**B**) ^11^C-PE2I and (**E**) ^11^C-DASB PET uptake change of a given seed ROI (*x*-axis) and the regression-derived association between its FC to target regions and PET uptake in the respective 137 target regions (*y*-axis). Illustration of the results shown for regions of highest (hotspot) and lowest (coldspot) ^11^C-PE2I (**C**, **D**) and ^11^C-DASB (**F**, **G**) PET uptake changes. For each of the 138 seed ROIs, a linear regression model was fitted using baseline group-average FC values between the seed and its 137 target ROIs (independent variable) to predict PET uptake changes in the corresponding targets (dependent variable), yielding one *β*-value per seed. This procedure produced 138 *β*-values per PET modality. To assess whether longitudinal PET changes in a region relate to its influence on brain-wide FC–PET coupling, Spearman's correlation was applied between PET uptake change in each seed ROI and its corresponding *β*-value. Each data point represents one seed ROI (*n* = 138). All *P*-values were FDR-corrected to account for multiple comparisons.

Longitudinal change covariance matrices of ^11^C-PE2I PET (Fig. 5A) and ^11^C-DASB PET (Fig. 5B) were obtained for 15 PD patients with follow-up data. Linear regressions revealed a significant positive relationship between the PD group FC matrix at baseline (derived from 15 PD patients who had follow-up data) and both the ^11^C-PE2I (*β* = 0.402, *P* < 0.001, Fig. 5C) and ^11^C-DASB (*β* = 0.370, *P* < 0.001, Fig. 5D) PET change covariance matrices. The associations between baseline FC and PET change covariance remained significant when controlling for Euclidean distance between ROIs in the regression model (^11^C-PE2I PET: *β* = 0.314, *P* < 0.001; ^11^C-DASB PET: *β* = 0.273, *P* < 0.001). Furthermore, we found significant positive correlations between baseline FC and ^11^C-PE2I PET change covariance (*β*-values ranging from 0.166 to 0.576, *P* < 0.05) and between baseline FC and ^11^C-DASB PET change covariance (*β*-values ranging from 0.312 to 0.671, *P* < 0.05) within all eight brain networks (see Supplementary Fig. 4). The *P*-values shown in the figure were FDR-corrected to account for multiple comparisons. Steiger's *Z* indicated that the correlations between FC and ^11^C-PE2I PET change covariance were significantly stronger than the correlation between FC and ^11^C-DASB PET change covariance for the whole brain (*Z* = 2.497, *P* = 0.013), AN (*Z* = 12.539, *P* < 0.001) and the BN network (*Z* = 22.021, *P* < 0.001). Conversely, correlations were significantly weaker for ^11^C-PE2I PET covariance than for ^11^C-DASB PET covariance in the VN (*Z* = −18.880, *P* < 0.001), SMN (*Z* = −9.318, *P* < 0.001), LN (*Z* = −16.131, *P* < 0.001), FPN (*Z* = −5.202, *P* < 0.001) and DMN networks (*Z* = −3.808, *P* < 0.001) (see Supplementary Table 3). Projecting longitudinal changes in ^11^C-PE2I and ^11^C-DASB PET uptake onto the functional network topology shows that the degree of longitudinal PET uptake changes tended to cluster within highly functionally connected regions (Fig. 6A).

### Longitudinal changes in PET uptake correlates with FC–PET associations in target ROIs

We found a significant positive correlation between the longitudinal changes in PET uptake across seed ROIs and their correspondent regression-derived *β*-value, representing the relationship between longitudinal changes in PET uptake in the target ROI and their FC to the seed ROI for both ^11^C-PE2I PET (*r* = 0.628, *P* < 0.001, Fig. 6B) and ^11^C-DASB PET (*r* = 0.560, *P* < 0.001, Fig. 6E). Positive correlations were found between the longitudinal changes in PET uptake in hotspots and their FC to the target ROI (Fig. 6C and F), while negative correlations were observed for cold spots (Fig. 6D and G). The *P*-values shown in the figure were FDR-corrected to account for multiple comparisons.

### Association between FC and PET uptake is correlated with clinical measures

**Figure 7:**
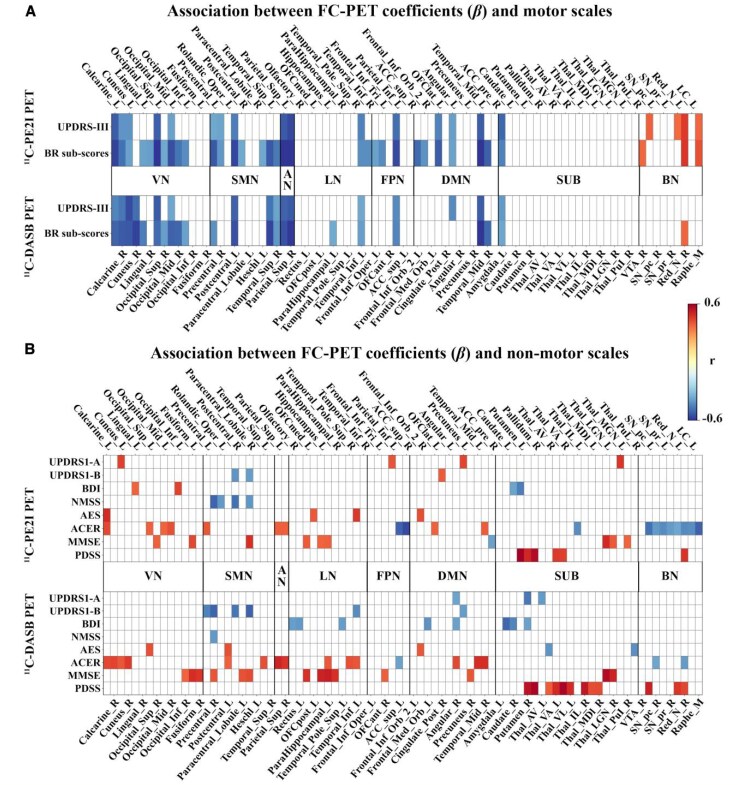
**Brain regions in which the association between FC and PET uptake is correlated with motor (A) and non-motor (B) scales in patients with PD.** For each patient (*N* = 30) and each tracer, FC–PET association maps were generated by performing linear regression at each seed ROI, with the vectorised FC to 137 target ROIs as the independent variable and the corresponding PET uptake as the dependent variable. This resulted in a 138-element *β*-vector per patient representing FC–PET coupling across the brain. Spearman's correlation was then used to identify seed regions where these *β*-values were significantly associated with clinical symptom severity scores (motor: MDS-UPDRS-III; non-motor: NMSS, BDI, AES, etc.). The figure only shows brain regions with FDR-corrected *P* < 0.05. Abbreviations: UPDRS = Unified Parkinson's disease rating scale; BR sub-score = bradykinesia-rigidity sub-score; BDI = Beck's depression inventory; NMSS = Non-Motor Symptoms Scale; AES = Apathy Evaluation Scale; ACER = Addenbrooke's Cognitive Examination Revised; MMSE = Mini-mental State Examination; PDSS = Parkinson's disease sleep scale; VN = Visual Network; SMN = Sensorimotor Network; AN = Attention Network; LN = Limbic Network; FPN = Frontoparietal Network; DMN = Default Mode Network; SUB = Subcortical Network; BN = Brainstem Network; Abbreviations for brain regions shown in the figure are listed in Supplementary Table 1.

We found that both motor and non-motor scales were correlated with the association between FC and PET uptake (i.e. FC–PET *β*-values) (Fig. 7, also see Supplementary Table 4 and 5, and 6). Specifically, for motor scales, the motor component of the MDS-UPDRS-III score correlated with the FC–PET *β*-values in 20/138 ROIs for ^11^C-PE2I and in 16/138 ROIs for ^11^C-DASB. Furthermore, the bradykinesia-rigidity sub-score correlated with the FC–PET *β*-values in 34/138 ROIs for ^11^C-PE2I and 23/138 ROIs for ^11^C-DASB. However, no significant results were found for the tremor sub-score. Regarding non-motor scales, the non-motor component of the MDS-UPDRS (UPDRS-IA and UPDRS-IB), BDI, NMSS, AES, ACER, MMSE and PDSS scores correlated with the FC–PET *β*-values in 4/138, 3/138, 4/138, 4/138, 4/138, 20/138, 10/138 and 6/138 ROIs for ^11^C-PE2I and in 3/138, 6/138, 8/138, 1/138, 5/138, 17/138, 14/138 and 12/138 ROIs for ^11^C-DASB, respectively.

Longitudinally, we found that changes in both motor and non-motor scales were correlated with the association between baseline FC and changes in PET uptake (see Supplementary Fig. 5, Supplementary Fig. 6 and Supplementary Table 7 for the ACER sub-score analysis).

## Discussion

The main findings of the current study were, firstly, that in patients with PD, higher FC between two given brain regions is associated with stronger interregional homogeneity of dopamine and serotonin levels as assessed by ^11^C-PE2I and ^11^C-DASB PET. Secondly, we found that highly functionally connected brain regions show similar longitudinal changes in ^11^C-PE2I and ^11^C-DASB PET uptake in patients with PD. Thirdly, the association between PET uptake in target ROIs and their FC to the seed region was correlated with PD motor and non-motor severity across different brain regions, depending on the neurotransmitter system evaluated.

To the best of our knowledge, this is the first study to evaluate the association between the spatial distribution of dopamine and serotonin and FC in patients with PD. Our first finding confirms our hypothesis that dopamine and serotonin levels covary among brain regions that are highly functionally connected, suggesting that there is a correspondence between the spatial distribution of neurotransmitters and the FC pattern. Notably, we observed stronger correlations between FC and dopamine covariance across the whole brain and within the SMN, LN, FPN, SUB and BN networks. These strong associations highlight the important role of dopaminergic modulation in maintaining and regulating the FC within these networks. This finding aligns with the fact that dopamine regulates motor control, emotion regulation and cognition functions.^43,44^ Conversely, in the VN, the correlation was more pronounced between FC and serotonin covariance. Additionally, this corresponds with a previous study suggesting a significant role for serotonin in mediating visual hallucinations associated with cognitive dysfunction through the VN in PD.^45^ These differential correlations across brain networks underscore the complexity of neurotransmitter interactions and their relationships with FC, highlighting that dopamine and serotonin play distinct roles in various brain networks. Importantly, associations between FC and PET covariance remained significant after controlling for Euclidean distance, suggesting that they are not solely driven by spatial proximity.

Longitudinally, we observed that brain regions that were highly functionally connected at baseline displayed similar longitudinal changes in ^11^C-PE2I and ^11^C-DASB PET uptake at follow-up, echoing our cross-sectional findings in PD. Again, there were network-specific differences in the strength of correlations between FC and the change covariances of ^11^C-PE2I and ^11^C-DASB PET. Our findings suggest that degeneration of the dopamine system over time has a stronger relationship with FC at the whole-brain level and within the AN and BN networks than does degeneration of the serotonin system. This observation is consistent with the fact that dopamine depletion is thought to be central in PD, especially the progressive loss of dopaminergic neurons in the substantia nigra.^46^ Although the primary clinical focus has long been on motor symptoms, there is increasing recognition of the importance of studying non-motor symptoms in patients with PD.^9,47^ The stronger correlation observed between FC and ^11^C-DASB PET change covariance compared with FC and ^11^C-PE2I PET change covariance in the VN, LN and DMN underscores serotonin's greater significance in emotional and self-referential processing compared to dopamine.^48,49^ This aligns with the concept that serotonin dysfunction in patients with PD is associated with the development of non-motor symptoms.^50^

We found that the predictive ability of FC, as indicated by regression-derived *β*-values, for the level of PET uptake in target ROIs was modulated by the level of PET uptake in the seed ROI. Specifically, for seeds with high PET uptake (i.e. hotspot), higher FC was associated with higher PET uptake in target ROIs (i.e. positive *β*-value). In contrast, for seeds with low PET uptake (i.e. cold spot), higher FC was associated with lower PET uptake in target ROIs (i.e. negative *β*-value). Furthermore, we observed that the rate of PET uptake decline in a given target brain region can be predicted by combining its baseline FC with that of the seed regions and the rate of PET uptake decline in those seed regions. These findings suggest that higher FC either enhances or diminishes the neurotransmitter activity in connected regions based on the initial state of the seed.

We found that the association between FC and PET uptake correlates with motor severity in patients with PD. This correlation was observed in more brain regions for ^11^C-PE2I PET compared with ^11^C-DASB PET, suggesting that these two radioligands differ in their sensitivities and specificities when assessing brain regions affected by PD. The correlations observed for both radioligands align with the fact that both the dopaminergic and serotonergic systems are affected in PD,^51^ though they vary in the degree to which they are disrupted. Our findings are consistent with the current understanding that the dopaminergic system plays a more significant role than the serotonergic system in the development of motor symptoms.^52^ However, it is worth mentioning that there was a lack of correlation between motor scales and FC–PET *β*-values for ^11^C-PE2I PET in the striatum. One possible explanation is that the striatum, particularly the putamen, is not a functionally uniform structure; different subregions are involved in distinct aspects of motor control.^53^ Subdividing the putamen into anterior and posterior regions may reveal more accurate correlations with motor scales. Additionally, the limited variability in striatal ^11^C-PE2I binding may have restricted our ability to detect associations with clinical measures. More broadly, floor and ceiling effects may influence both the imaging and clinical measures used. For example, DAT availability may show less progressive decline in PD compared to serotonergic markers, and clinical scales may lack the sensitivity to detect subtle variations, particularly in motor symptoms, within a moderately affected cohort.^54,55^

Furthermore, the observed correlation in more brain regions for the bradykinesia-rigidity sub-score than for the total UPDRS-III score suggests that bradykinesia and rigidity are more sensitive to alterations in neurotransmitter systems and functional connectomes in patients with PD. This finding is in agreement with our previous study, which showed that both striatal ^11^C-PE2I and ^18^F-DOPA uptakes are more strongly correlated with the bradykinesia-rigidity sub-score than with UPDRS-III.^16^ Similarly, Kerstens *et al*. using ^18^F-FE-PE2I PET, found that DAT availability in the motor striatum is more strongly correlated with bradykinesia and rigidity than with UPDRS-III.^13^ Increasing evidence suggests that PD is not a single entity, rather it encompasses various clinical subtypes.^56,57^ Based on clinical symptoms, some patients with PD are tremor-dominant, while others exhibit more pronounced bradykinesia-rigidity.^58^ The total UPDRS-III score provides a comprehensive assessment of motor symptoms in patients with PD, including tremor, bradykinesia-rigidity and postural abnormalities.^59^ However, this broad assessment may dilute the effects of specific symptoms, resulting in weaker correlations compared with focusing solely on bradykinesia-rigidity sub-scores. Taken together, our findings suggest that future studies might benefit from focusing on clinical motor sub-scores, rather than composite scores, to achieve a more specific and accurate evaluation of associations between different symptoms and neuroimaging measures.

Previous studies have demonstrated a lack of correlation between ^11^C-DASB binding and both the total score and sub-scores of the UPDRS-III, suggesting that the serotonergic system may not be involved in the development of motor symptoms in PD.^21,60^ In contrast, we found that the FC–PET *β*-values for ^11^C-DASB (i.e. the association between FC and ^11^C-DASB PET uptake) were significantly correlated with motor severity in patients with PD. This suggests that the development of motor symptoms in PD is not solely due to the degeneration of the dopaminergic system but may also arise from the functional interaction of other neurotransmitter systems and brain networks. Altogether, our findings highlight the complexity of the underlying pathology of PD and underline the value of using multimodal imaging techniques to study the disease.

We also found that the association between FC and PET uptake correlates with non-motor scales in patients with PD. However, in contrast to motor scales, this correlation was observed in more brain regions for ^11^C-DASB PET compared with ^11^C-PE2I PET. Our results align with the prevailing view that the serotonergic system has a greater influence than the dopaminergic system in the emergence of non-motor symptoms in patients with PD.^61,62^ Non-motor symptoms, such as depression, apathy, cognitive impairment and sleep disorders are often overshadowed by the more overt motor symptoms but significantly affect patients with PD quality of life.^63^ Our findings underscore that both dopaminergic and serotonergic systems are involved in the development of non-motor symptoms in PD, but they do so via distinct networks and mechanisms.

Longitudinally, significant correlations were found between changes in motor severity and the association between baseline FC and changes in PET uptake. These correlations were observed in more brain regions for ^11^C-PE2I than for ^11^C-DASB, suggesting that dopaminergic dysfunction is more responsible for motor function decline as the disease progresses than serotonergic dysfunction in PD, echoing our cross-sectional findings. For non-motor scales, longitudinal changes in the BDI were positively correlated with the association between baseline FC and changes in ^11^C-PE2I PET uptake in brain regions within the subcortical and brainstem networks. Conversely, longitudinal changes in the BDI were negatively correlated with the association between baseline FC and changes in ^11^C-DASB PET uptake in brain regions within the SNM, AN, LN, FPN and DMN networks. Our results align with a previous study that found that dopamine and serotonin have contrasting effects on brain networks.^64^

The correlations between FC, PET uptake and clinical measures observed in this study could have important therapeutic and clinical implications for PD. Traditional treatments for patients with PD often target dopamine levels exclusively, and the classic motor symptoms of bradykinesia and rigidity respond well to dopaminergic therapies.^65^ However, dopaminergic drugs are often ineffective against tremor and non-motor symptoms, and they may also lead to off-target effects and side effects, such as dyskinesias.^66^ In this study, we observed that the serotonergic system, traditionally thought to be associated with non-motor symptoms, also correlates with motor severity in PD when interacting with FC. This finding highlights the potential influence of factors such as the mood on patients with PD at the time of assessment, which can affect motor severity ratings measured by the UPDRS-III.^67^ Consequently, treatments targeting both dopamine and serotonin may be more effective and comprehensive in managing PD symptoms than those focusing solely on dopamine. Furthermore, the region- and network-specific relationships we observed between the dopaminergic and serotonergic systems, FC and specific symptoms could help clinicians develop more customized therapeutic strategies for patients with PD. For example, brain regions within the SUB network are thought to be predominantly associated with bradykinesia and rigidity, and targeted interventions, such as dopaminergic-replacement therapies and modulation of FC within this network, could be used to address these particular symptoms. Indeed, this treatment strategy has been applied in deep brain stimulation to alleviate motor symptoms in patients with PD by modulating FC within specific subnetworks, achieving good therapeutic effects.^68^ Similarly, if a specific brain region or network is identified as being primarily associated with non-motor symptoms such as visual hallucinations, mood and sleep problems, treatments should target the serotonergic system.

This study has some limitations. Firstly, due to the unavailability of ^11^C-PE2I and ^11^C-DASB PET data for HCs, we could not determine whether the spatial distribution of these neurotransmitters was associated with FC in HCs, or if such an association was exclusive to patients with PD. Secondly, the current study focuses strictly on FC, which is largely matched by SC as assessed by diffusion tensor imaging (DTI), but not entirely.^69^ Future studies should combine both rs-fMRI and DTI in order to assess the joint contribution of FC and SC to the spatial distribution of neurotransmitters, thereby enhancing our understanding of the association between the spatial distribution of neurotransmitter and brain connectivity. Thirdly, while we used an atlas-based parcellation to ensure consistency across participants and alignment with prior multimodal imaging studies, we acknowledge that this approach may not fully capture the functional heterogeneity within certain networks. Future studies may benefit from incorporating data-driven network assignment methods to improve spatial specificity and better account for individual variability in functional organization. Fourthly, although the patients with PD recruited in our study were in the early stages of the disease, and the findings on brain atrophy in early-stage PD are inconsistent,^70,71^ future studies should consider grey matter volume when evaluating rs-fMRI and PET data to account for any potential impact on these measures. Fifthly, due to the small sample size, the clinical scales used may not be sensitive enough to detect subtle variations in clinical outcomes, and the homogeneity of our study population may limit the generalizability of our findings. It is essential to explore these relationships between FC–PET and clinical measures further in future studies with larger sample sizes and more diverse cohorts to validate our findings. Lastly, although the present study focused on examining dopaminergic and serotonergic systems independently, we acknowledge that the relative balance between these neurotransmitters may provide additional insights into the functional organization of the PD brain. It would be valuable for future research to investigate this interplay more explicitly, including region-specific dopamine-to-serotonin ratios and their combined effects on FC.

In summary, the current study demonstrates that highly functionally connected brain regions in patients with PD exhibit similar dopamine and serotonin levels, as well as similar changes in dopamine and serotonin levels over time. Our findings suggest a significant correspondence between the patterns of FC and the spatial distribution of dopamine and serotonin, which is associated with several features of the disease. An understanding of the complex interaction between dopaminergic and serotonergic systems and functional networks may lead to more comprehensive therapeutic approaches to treat the motor and non-motor symptoms of PD.

## Supplementary Material

fcaf308_Supplementary_Data

## Data Availability

Data on patients with PD are available from the authors upon reasonable request. The codes used in this study are provided as .*py* files in the Supplementary material.
