# Supplementary material for: Mapping the distribution of neurotransmitters to resting-state functional connectivity in Parkinson’s disease
Source: Brain Commun. 2025 Sep 9;7(5):fcaf308. doi: 10.1093/braincomms/fcaf308 (PMC12418387; doi:10.1093/braincomms/fcaf308)

Supplementary Table 1: The 138 regions-of-interest (ROIs) from the automated anatomical labeling 3 (AAL3) atlas are grouped into 8 networks.

Network	AAL3 id		AAL3 label	AAL3 label fullname
Visual network (VN)	47	48	Calcarine	Calcarine fissure
	49	50	Cuneus	Cuneus
	51	52	Lingual	Lingual gyrus
	53	54	Occipital_Sup	Superior occipital gyrus
	55	56	Occipital_Mid	Middle occipital gyrus
	57	58	Occipital_Inf	Inferior occipital gyrus
	59	60	Fusiform	Fusiform gyrus
Sensorimotor network (SMN)	1	2	Precentral	Precentral gyrus
	13	14	Rolandic_Oper	Rolandic operculum
	15	16	Supp_Motor_Area	Supplementary motor area
	61	62	Postcentral	Postcentral gyrus
	73	74	Paracentral_Lobule	Paracentral lobule
	83	84	Heschl	Heschl's gyrus
	85	86	Temporal_Sup	Superior temporal gyrus
Attention network (AN)	33	34	Insula	Insula
	37	38	Cingulate_Mid	Middle cingulate & paracingulate gyri
	63	64	Parietal_Sup	Superior parietal gyrus
	67	68	SupraMarginal	SupraMarginal gyrus
Limbic network (LN)	17	18	Olfactory	Olfactory cortex
	23	24	Rectus	Gyrus rectus
	25	26	OFCmed	Medial orbital gyrus
	29	30	OFCpost	Posterior orbital gyrus
	41	42	Hippocampus	Hippocampus
	43	44	ParaHippocampal	Parahippocampal gyrus
	87	88	Temporal_Pole_Sup	Temporal pole
	91	92	Temporal_Pole_Mid	Temporal pole: middle temporal gyrus
	93	94	Temporal_Inf	Inferior temporal gyrus
Frontoparietal network (FPN)	5	6	Frontal_Mid_2	Middle frontal gyrus
	7	8	Frontal_Inf_Oper	Inferior frontal gyrus, opercular part
	9	10	Frontal_Inf_Tri	Inferior frontal gyrus, triangular part
	27	28	OFCant	Anterior orbital gyrus
	65	66	Parietal_Inf	Inferior parietal gyrus
	155	156	ACC_sup	Anterior cingulate cortex
Default mode network (DMN)	3	4	Frontal_Sup_2	Superior frontal gyrus, dorsolateral
	11	12	Frontal_Inf_Orb_2	Inferior frontal gyrus, pars orbitalis
	19	20	Frontal_Sup_Medial	Superior frontal gyrus, medial
	21	22	Frontal_Med_Orb	Superior frontal gyrus, medial orbital
	31	32	OFClat	Lateral orbital gyrus
	39	40	Cingulate_Post	Posterior cingulate gyrus
	69	70	Angular	Angular gyrus
	71	72	Precuneus	Precuneus
	89	90	Temporal_Mid	Middle temporal gyrus

	151	152	ACC_sub	Anterior cingulate cortex, subgenual
	153	154	ACC_pre	Anterior cingulate cortex, pregenual
Subcortical network (SUB)	45	46	Amygdala	Amygdala
	75	76	Caudate	Caudate
	77	78	Putamen	Putamen
	79	80	Pallidum	Pallidum
	121	122	Thal_AV	Thalamus Anteroventral Nucleus
	123	124	Thal_LP	Lateral posterior
	125	126	Thal_VA	Ventral anterior
	127	128	Thal_VL	Ventral lateral
	129	130	Thal_VPL	Ventral posterolateral
	131	132	Thal_IL	Intralaminar
	135	136	Thal_MDm	Mediodorsal medial magnocellular
	137	138	Thal_MDl	Mediodorsal lateral parvocellular
	139	140	Thal_LGN	Lateral geniculate
	141	142	Thal_MGN	Medial Geniculate
	143	144	Thal_PuI	Pulvinar anterior
	145	146	Thal_PuM	Pulvinar medial
	147	148	Thal_PuA	Pulvinar lateral
	149	150	Thal_PuL	Pulvinar inferior
	157	158	N_Acc	Nucleus accumbens
Brainstem (BN)	159	160	VTA	Ventral tegmental area
	161	162	SN_pc	Substantia nigra, pars compacta
	163	164	SN_pr	Substantia nigra, pars reticulata
	165	166	Red_N	Red nucleus
	167	168	LC	Locus coeruleus
	169		Raphe_D	Raphe nucleus, dorsal
	170		Raphe_M	Raphe nucleus, median

Supplementary Table 2: ^{11}C-PE2I and ^{11}C-DASB PET hotspot and coldspot regions.

	Baseline		Change	
	**^{11}C-PE2I**	**^{11}C-DASB**	**^{11}C-PE2I**	**^{11}C-DASB**
**Hotspot**	Nucleus accumbens (R)	Dorsal raphe nucleus	Amygdala (L)	Thalamus: Ventral posterolateral (L)
**Coldspot**	Superior parietal gyrus (R)	Lateral orbital gyrus (R)	Nucleus accumbens (R)	Thalamus: Lateral posterior (R)

Supplementary Table 3: The Steiger's *Z* procedure was used to assess whether the correlation coefficient describing the relationship between functional connectivity and ^{11}C-PE2I PET covariance significantly differed from that describing the relationship between functional connectivity and ^{11}C-DASB PET covariance, over the whole brain as well as within the 8 functional networks. * Indicates p <0.05; ** Indicates p <0.001

**Networks**	**Baseline**		**Follow-up**	
	***Z*-score**	***P*-value**	***Z*-score**	***P*-value**
Whole brain	**2.858**	**0.004***	**2.497**	**0.013***
Visual network (VN)	**-13.704**	**<0.001****	**-18.880**	**<0.001****
Sensorimotor network (SMN)	**8.473**	**<0.001****	**-9.318**	**<0.001****
Attention network (AN)	-1.046	0.295	**12.539**	**<0.001****
Limbic network (LN)	**21.221**	**<0.001****	**-16.131**	**<0.001****
Frontoparietal network (FPN)	**3.502**	**<0.001****	**-5.202**	**<0.001****
Default mode network (DMN)	1.905	0.057	**-3.808**	**<0.001****
Subcortical network (SUB)	**16.069**	**<0.001****	1.271	0.203
Brainstem network (BN)	**36.750**	**<0.001****	**22.021**	**<0.001****

Supplementary Table 4: Brain regions in which the association between FC and PET uptake is correlated with motor scale in PD patients.

Network	Brain regions	Association between functional connectivity and ^{11}C-PE2I PET (*β*-values)				Association between functional connectivity and ^{11}C-DASB PET (*β*-values)			
		Total UPDRS-III scores		Bradykinesia-Rigidity sub-score		Total UPDRS-III scores		Bradykinesia-Rigidity sub-score	
		r	*p*	*r*	*p*	*r*	*p*	*r*	*p*
VN	Calcarine fissure (L)	-0.524	0.003	-0.576	0.001	-0.444	0.014	-0.507	0.004
	Calcarine fissure (R)	-0.409	0.025	-0.482	0.007	-0.470	0.009	-0.526	0.003
	Cuneus (L)	-0.410	0.024	-0.380	0.038	-0.556	0.001	-0.531	0.003
	Cuneus (R)					-0.490	0.006	-0.573	0.001
	Lingual gyrus (L)			-0.382	0.037			-0.458	0.011
	Lingual gyrus (R)			-0.387	0.034				
	Superior occipital gyrus (L)	-0.518	0.003	-0.606	<0.001	-0.528	0.003	-0.496	0.005
	Superior occipital gyrus (R)			-0.367	0.046			-0.387	0.034
	Middle occipital gyrus (L)	-0.382	0.037	-0.550	0.002	-0.375	0.041	-0.512	0.004
	Middle occipital gyrus (R)			-0.476	0.008			-0.505	0.004
	Inferior occipital gyrus (L)			-0.424	0.019			-0.381	0.038
SMN	Precentral gyrus (L)	-0.367	0.046	-0.531	0.003			-0.419	0.021
	Precentral gyrus (R)	-0.378	0.040	-0.406	0.026				
	Postcentral gyrus (L)	-0.456	0.011	-0.568	0.001	-0.513	0.004	-0.547	0.002
	Postcentral gyrus (R)			-0.362	0.050				
	Heschl's gyrus (L)			-0.369	0.045				
	Superior temporal gyrus (L)			-0.533	0.002	-0.474	0.008	-0.568	0.001
	Superior temporal gyrus (R)			-0.465	0.010	-0.379	0.039	-0.404	0.027
AN	Superior parietal gyrus (L)	-0.526	0.003	-0.596	0.001	-0.511	0.004	-0.547	0.002
	Superior parietal gyrus (R)	-0.562	0.001	-0.670	<0.001	-0.526	0.003	-0.588	0.001
LN	Parahippocampal gyrus (L)							-0.366	0.047
	Inferior temporal gyrus (L)	-0.450	0.013	-0.425	0.019	-0.370	0.044	-0.385	0.036
	Inferior temporal gyrus (R)			-0.426	0.019				

	Inferior frontal gyrus, opercular (L)			-0.384	0.036				
**FPN**	Inferior frontal gyrus, triangular (L)			-0.427	0.019				
	Inferior parietal gyrus (L)	-0.489	0.006	-0.560	0.001	-0.432	0.017	-0.441	0.015
	IFG pars orbitalis (L)			-0.493	0.006				
	IFG pars orbitalis (R)			-0.414	0.023				
**DMN**	Lateral orbital gyrus (L)	-0.446	0.013	-0.523	0.003				
	Angular gyrus (L)	-0.387	0.035	-0.383	0.037	-0.432	0.017		
	Middle temporal gyrus (L)	-0.524	0.003	-0.648	<0.001	-0.522	0.003	-0.636	<0.001
	Middle temporal gyrus (R)			-0.517	0.003			-0.498	0.005
**SUB**	Amygdala (L)	-0.504	0.005	-0.454	0.012	-0.403	0.027	-0.373	0.042
	Ventral tegmental area (R)			0.368	0.046				
	Substantia nigra, pars compacta (L)	0.380	0.038						
**BN**	Red nucleus (L)	0.363	0.049						
	Red nucleus (R)	0.456	0.011	0.468	0.009			0.375	0.041
	Raphe nucleus, median	0.374	0.042	0.415	0.023				

Supplementary Table 5: Brain regions in which the association between FC and ^{11}C-PE2I PET uptake is correlated with non-motor scale in PD patients.

Network	Brain regions	UPDRS-I A scores		UPDRS-I B scores		Beck Depression Inventory (BDI)		Non Motor Symptom Scale (NMSS)		Apathy Evaluation Scale (AES)		Addenbrooke's Cognitive Examination (ACE-R)		Mini-mental State Examination (MMSE)		PD Sleep Scale (PDSS)	
		r	*p*	*r*	*p*	*r*	*p*	*r*	*p*	*r*	*p*	*r*	*p*	*r*	*p*	*r*	*p*
VN	Calcarine fissure (L)									0.489	0.011	0.429	0.023				
	Cuneus (L)	0.442	0.019														
	Lingual gyrus (L)					0.377	0.048										
	Superior occipital gyrus (L)											0.382	0.045				
	Superior occipital gyrus (R)													0.383	0.044		
	Middle occipital gyrus (L)											0.377	0.048				
	Middle occipital gyrus (R)											0.417	0.027				
	Inferior occipital gyrus (L)					0.44	0.019										
	Fusiform gyrus (L)													0.43	0.022		
SMN	Precentral gyrus (L)											0.403	0.034				
	Precentral gyrus (R)							-0.507	0.01								
	Rolandic operculum (L)							-0.4	0.048								
	Postcentral gyrus (R)			-0.412	0.033			-0.48	0.015								
	Paracentral lobule (R)			-0.393	0.043			-0.403	0.046					0.481	0.01		

**AN**	Superior parietal gyrus (L)											0.385	0.043				
	Superior parietal gyrus (R)											0.388	0.042				
**LN**	Medial orbital gyrus (L)													0.391	0.04		
	Posterior orbital gyrus (L)									0.397	0.045						
	Hippocampus (L)													0.376	0.048		
	Parahippocampal gyrus (L)													0.38	0.046		
	Inferior temporal gyrus (L)									0.461	0.018						
**FPN**	Inferior parietal gyrus (L)	0.406	0.032														
	Anterior cingulate cortex (L)											-0.509	0.006				
	Anterior cingulate cortex (R)											-0.561	0.002				
**DMN**	IFG pars orbitalis (R)									0.422	0.032						
	Lateral orbital gyrus (L)											0.381	0.046				
	Posterior cingulate gyrus (R)			0.392	0.043												
	Precuneus (L)	0.433	0.021														
	Middle temporal gyrus (R)											0.393	0.038				
	Anterior cingulate cortex, pregenual (R)													-0.381	0.045		
**SUB**	Caudate nucleus (R)					-0.378	0.047										
	Lenticular nucleus, putamen (L)					-0.455	0.015									0.626	<0.001

	Lenticular nucleus, putamen (R)															0.499	0.009
	Lenticular nucleus, pallidum (R)															0.567	0.003
	Ventral anterior (L)															0.455	0.019
	Ventral anterior (R)															0.449	0.021
	Intralaminar (L)											-0.424	0.024				
	Lateral geniculate (L)													0.497	0.007		
	Lateral geniculate (R)													0.397	0.037		
	Medial Geniculate (L)	0.468	0.012														
	Pulvinar anterior (R)													0.375	0.049		
	Substantia nigra, pars compacta (L)											-0.48	0.01				
	Substantia nigra, pars compacta (R)											-0.402	0.034				
	Substantia nigra, pars reticulata (L)											-0.428	0.023				
	Substantia nigra, pars reticulata (R)											-0.397	0.037				
**BN**	Red nucleus (L)											-0.374	0.05				
	Red nucleus (R)											-0.433	0.021			0.47	0.015
	Locus coeruleus (L)											-0.425	0.024				
	Raphe nucleus, median											-0.513	0.005				

Supplementary Table 6: Brain regions in which the association between FC and ^{11}C-DASB PET uptake is correlated with non-motor scale in PD patients.

Network	Brain regions	UPDRS-I A scores		UPDRS-I B scores		Beck Depression Inventory (BDI)		Non Motor Symptom Scale (NMSS)		Apathy Evaluation Scale (AES)		Addenbrooke's Cognitive Examination (ACE-R)		Mini-mental State Examination (MMSE)		PD Sleep Scale (PDSS)	
		r	***p***	***r***	***p***	***r***	***p***	***r***	***p***	***r***	***p***	***r***	***p***	***r***	***p***	***r***	***p***
**VN**	Calcarine fissure (L)											0.429	0.023				
	Calcarine fissure (R)											0.441	0.019				
	Cuneus (L)											0.401	0.035				
	Cuneus (R)											0.455	0.015				
	Superior occipital gyrus (L)									0.417	0.034						
	Inferior occipital gyrus (R)													0.375	0.049		
	Fusiform gyrus (L)													0.485	0.009		
	Fusiform gyrus (R)													0.466	0.012		
**SMN**	Precentral gyrus (L)			-0.447	0.02												
	Precentral gyrus (R)			-0.511	0.006			-0.402	0.046					0.404	0.033		
	Postcentral gyrus (L)									0.415	0.035	0.382	0.045				
	Postcentral gyrus (R)			-0.473	0.013												
	Paracentral lobule (L)													0.408	0.031		
	Paracentral lobule (R)			-0.513	0.006									0.423	0.025		
	Superior temporal gyrus (L)											0.416	0.028				

**AN**	Superior parietal gyrus (L)											0.502	0.006				
	Superior parietal gyrus (R)											0.471	0.011				
**LN**	Olfactory cortex (R)					-0.386	0.043										
	Gyrus rectus (L)					-0.401	0.034										
	Medial orbital gyrus (L)													0.478	0.01		
	Hippocampus (L)													0.51	0.006		
	Parahippocampal gyrus (L)											0.396	0.037	0.521	0.004		
	Parahippocampal gyrus (R)													0.482	0.009		
	Temporal pole (L)					-0.39	0.04										
	Temporal pole (R)											0.415	0.028				
	Inferior temporal gyrus (L)			-0.43	0.025							0.404	0.033				
**FPN**	Anterior orbital gyrus (R)													0.388	0.041		
	Anterior cingulate cortex (L)											-0.381	0.045				
**DMN**	IFG pars orbitalis (R)									0.402	0.042						
	Superior frontal gyrus, medial orbital (L)					-0.416	0.028										
	Angular gyrus (R)	-0.376	0.049			-0.382	0.045					0.427	0.023				
	Precuneus (L)			-0.393	0.043												
	Precuneus (R)													0.398	0.036		

	Middle temporal gyrus (L)											0.487	0.009				
	Middle temporal gyrus (R)											0.484	0.009				
	Caudate nucleus (L)					-0.503	0.006										
	Caudate nucleus (R)					-0.444	0.018										
	Lenticular nucleus, putamen (R)	-0.468	0.012			-0.382	0.045									0.542	0.004
	Lenticular nucleus, pallidum (R)															0.581	0.002
	Thalamus, Anteroventral Nucleus (L)	-0.395	0.038														
	Thalamus, Anteroventral Nucleus (R)									-0.412	0.036					0.39	0.049
	Ventral anterior (L)															0.494	0.01
**SUB**	Ventral anterior (R)															0.582	0.002
	Ventral lateral (L)															0.491	0.011
	Intralaminar (R)															0.537	0.005
	Mediodorsal lateral parvocellular (L)															0.42	0.033
	Mediodorsal lateral parvocellular (R)															0.423	0.031
	Lateral geniculate (L)													0.563	0.002		
	Lateral geniculate (R)													0.508	0.006		
	Pulvinar inferior (R)									-0.421	0.032						
**BN**	Substantia nigra, pars compacta (L)															0.514	0.007

	Substantia nigra, pars compacta (R)											-0.374	0.05				
	Red nucleus (L)															0.465	0.017
	Red nucleus (R)											-0.394	0.038			0.432	0.028

Supplementary Table 7: Brain regions in which the association between FC and PET uptake is correlated with ACER sub-scale in PD patients.

Network	Brain regions	Association between functional connectivity and ^{11}C-PE2I PET (*β*-values)								Association between functional connectivity and ^{11}C-DASB PET (*β*-values)							
		Verbal Fluency (acetflu)		Language (acetlan)		Attention and Memory (acetmem)		Visuospatial Ability (acetvis)		Verbal Fluency (acetflu)		Language (acetlan)		Attention and Memory (acetmem)		Visuospatial Ability (acetvis)	
		r	***p***	***r***	***p***	***r***	***p***	***r***	***p***	***r***	***p***	***r***	***p***	***r***	***p***	***r***	***p***
**VN**	Calcarine fissure and surrounding cortex (R)													0.454	0.015		
	Cuneus (R)													0.390	0.040		
	Middle occipital gyrus (L)							0.389	0.041							0.424	0.025
	Inferior occipital gyrus (L)							0.465	0.013							0.482	0.009
	Fusiform gyrus (R)							-0.378	0.047							-0.400	0.035
**SMN**	Precentral gyrus (L)															0.385	0.043
**AN**	Superior parietal gyrus (L)							0.519	0.005	0.388	0.041					0.589	0.001
	Superior parietal gyrus (R)							0.494	0.008							0.4	0.0

																41	19
	Supramarginal gyrus (L)			-0.375	0.049												
	Supramarginal gyrus (R)			-0.456	0.015							-0.433	0.021				
**LN**	Gyrus rectus (L)							0.413	0.029								
	Parahippocampal gyrus (L)							-0.449	0.017					0.383	0.044		
	Parahippocampal gyrus (R)							-0.489	0.008					0.396	0.037	-0.415	0.028
	Temporal pole: middle temporal gyrus (L)							-0.405	0.032								
	Inferior temporal gyrus (L)							0.375	0.049								
**FPN**	Inferior parietal gyrus, excluding supramarginal and angular gyri (L)							0.564	0.002							0.551	0.002
	Anterior cingulate cortex, supracallosal (L)							-0.611	0.001							-0.604	0.001
	Anterior cingulate cortex, supracallosal (R)	-0.420	0.026					-0.442	0.019								
**DMN**	Superior frontal gyrus, dorsolateral (L)							0.449	0.017							0.416	0.028
	Lateral orbital gyrus (L)							0.527	0.004								
	Posterior cingulate gyrus (R)													0.378	0.048		

	Precuneus (R)													0.399	0.035		
	Middle temporal gyrus (L)									0.385	0.043						
	Middle temporal gyrus (R)							0.500	0.007							0.403	0.034
	Anterior cingulate cortex, pregenual (R)					-0.465	0.013										
	Lenticular nucleus, putamen (L)															-0.533	0.003
	Lenticular nucleus, pallidum (L)															-0.431	0.022
	Ventral lateral (R)					0.408	0.031	-0.377	0.048								
	Ventral posterolateral (L)							-0.417	0.027								
**SUB**	Ventral posterolateral (R)					0.444	0.018	-0.421	0.025					0.386	0.042		
	Intralaminar (L)							-0.452	0.016							-0.446	0.017
	Intralaminar (R)					0.386	0.043	-0.424	0.025								
	Mediodorsal medial magnocellular (R)							-0.399	0.036								
	Mediodorsal lateral parvocellular (L)							-0.380	0.046								
	Mediodorsal lateral parvocellular (R)							-0.390	0.040								

	Lateral geniculate (L)			0.410	0.030			-0.526	0.004							-0.476	0.010
	Medial Geniculate (R)							-0.470	0.012								
	Pulvinar anterior (R)					0.401	0.034	-0.550	0.002							-0.442	0.019
	Pulvinar medial (R)							-0.442	0.019								
	Pulvinar lateral (R)							-0.387	0.042								
	Pulvinar inferior (R)					0.469	0.012										
	Nucleus accumbens (R)															-0.377	0.048
BN	Ventral tegmental area (R)							-0.455	0.015								
	Substantia nigra, pars compacta (L)					-0.478	0.010										
	Substantia nigra, pars compacta (R)							-0.408	0.031								
	Substantia nigra, pars reticulata (L)							-0.413	0.029							-0.412	0.030
	Red nucleus (L)							-0.401	0.034								
	Red nucleus (R)							-0.417	0.027								
	Locus coeruleus (R)			-0.433	0.021												
	Raphe nucleus, median							-0.435	0.021							-0.	0.0

																420	26

Supplementary Figure 1: **Analysis pipeline for assessing the association between group-average FC of a seed ROI and PET uptakes in target ROIs.** All ROIs were vectorised and rank-ordered by group-average PET uptake. For each seed ROI, group-average FC with the remaining ROIs (target regions) was computed. PET uptake values in the target ROIs were then regressed onto these FC measures. This procedure was repeated for all seed ROIs, yielding a sequence of β-values. Abbreviations: PET = positron emission tomography; ROI = region of interest; FC = functional connectivity; BP_{ND} = non-displaceable binding potential for ^{11}C-PE2I and ^{11}C-DASB PET

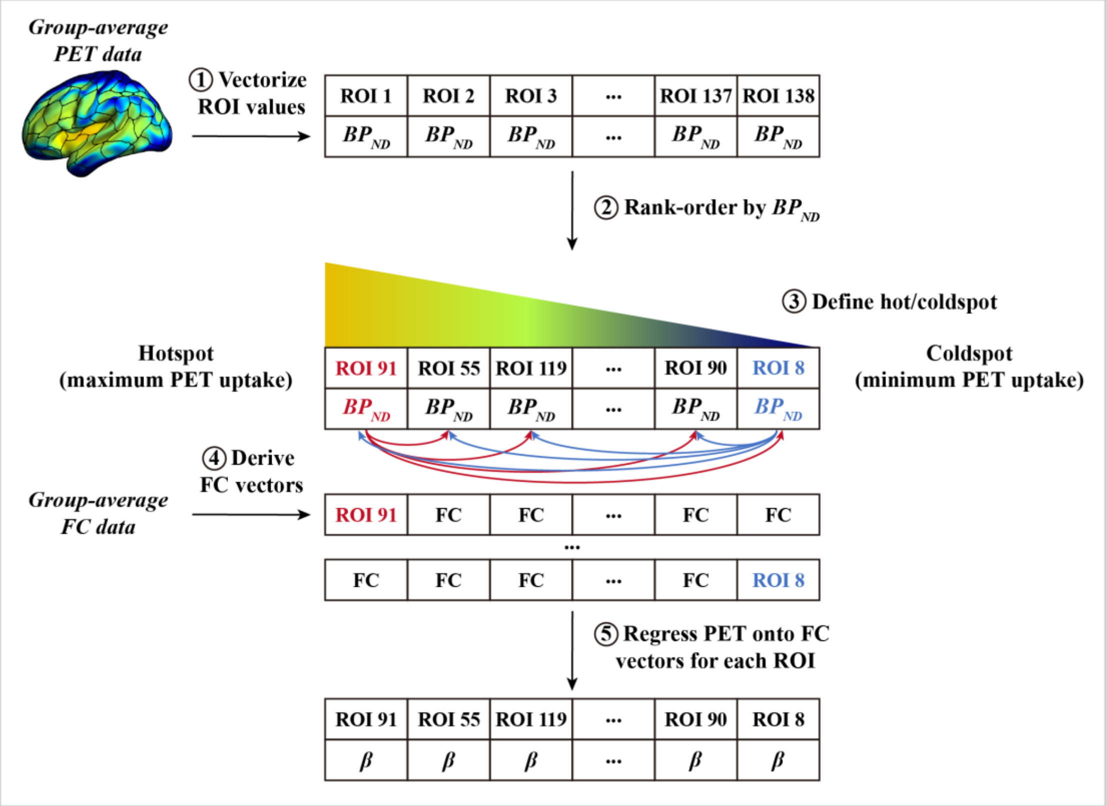

Supplementary Figure 2: Analysis pipeline for assessing the association between FC of a seed ROI and PET uptake in target ROIs for each PD patient and evaluating how this relates to disease severity. Linear regression was performed for each seed ROI, with the vectorised FC to the remaining 137 target ROIs as the independent variable and the corresponding vectorised PET uptake as the dependent variable. This yielded a 138-element β-vector representing the FC–PET association across the whole brain. Spearman's correlation was then used to identify brain regions where these FC–PET coefficients were significantly associated with PD symptom severity. Abbreviations: PET = positron emission tomography; ROI = region of interest; FC = functional connectivity; BP_{ND} = non-displaceable binding potential for ^{11}C-PE2I and ^{11}C-DASB PET; UPDRS = Unified Parkinson's Disease Rating Scale.

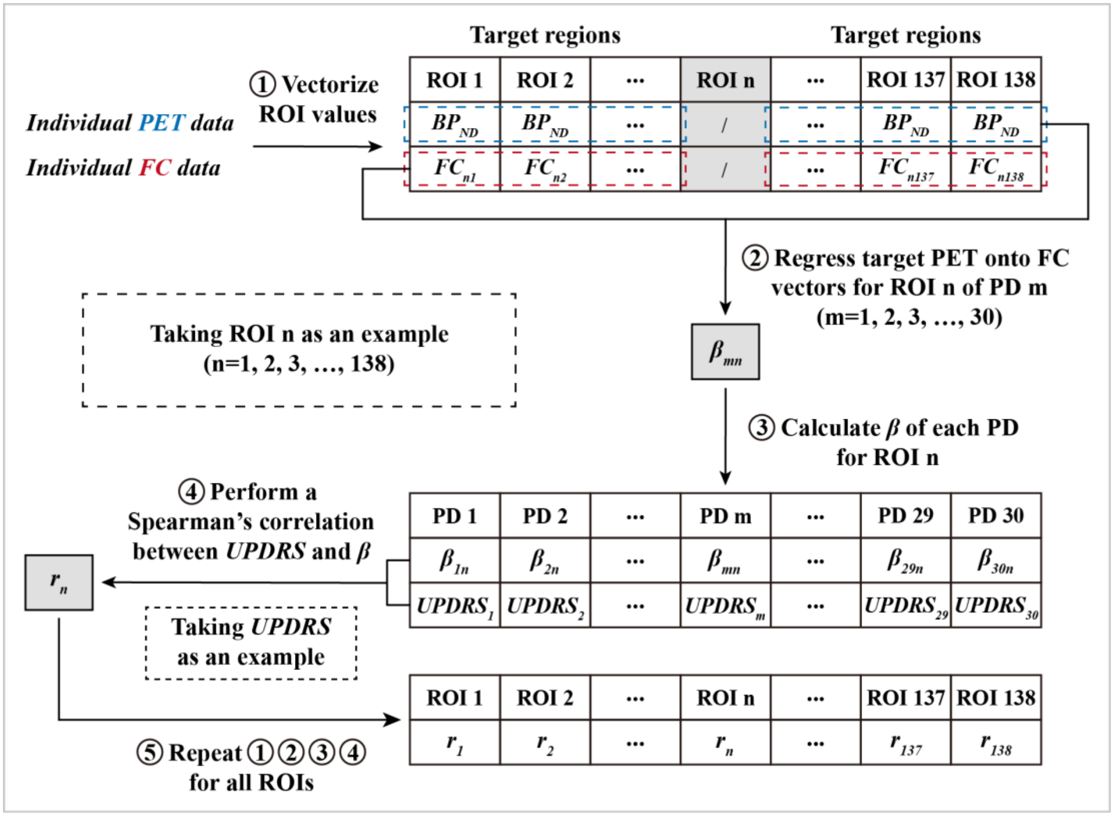

Supplementary Figure 3: Analysis pipeline for assessing the association between baseline FC of a seed ROI and changes in PET uptakes in target ROIs for each PD patient and evaluating how this relates to changes in disease severity. Linear regression was performed for each seed ROI, with the vectorised baseline FC to the remaining 137 target ROIs as the independent variable and the corresponding vectorised PET uptake changes (follow-up - baseline) as the dependent variable. This yielded a 138-element β-vector representing the FC–PET association across the whole brain. Spearman's correlation was then used to identify brain regions where these FC–PET coefficients were significantly associated with changes in PD symptom severity. Abbreviations: PET = positron emission tomography; ROI = region of interest; FC = functional connectivity; BP_{ND} = non-displaceable binding potential for ^{11}C-PE2I and ^{11}C-DASB PET; UPDRS = Unified Parkinson's Disease Rating Scale.

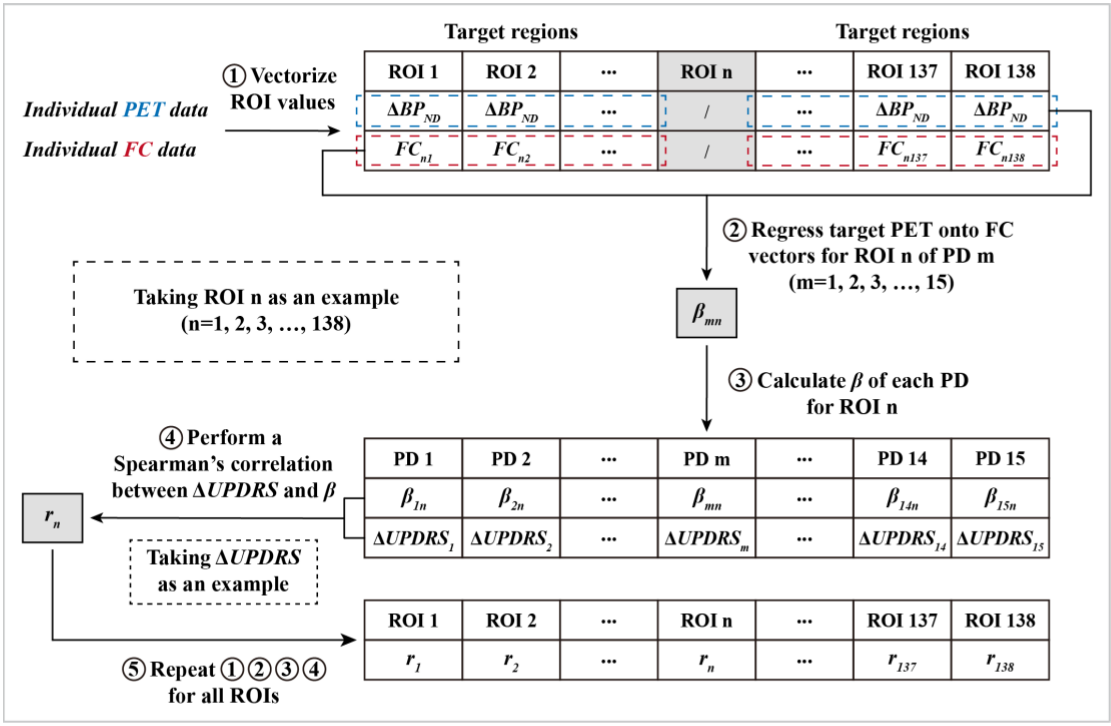

Supplementary Figure 4: **Association between baseline functional connectivity and PET change covariance in PD patients at follow-up (N = 15).** Scatterplots showing the association between group-average functional connectivity and (A) ^{11}C-PE2I PET change covariance and (B) ^{11}C-DASB PET change covariance for the 8 brain networks. Statistical analysis was performed using linear regression, with vectorised baseline FC as the independent variable and vectorised PET change covariance as the dependent variable. Each data point in the scatterplots represents a pairwise comparison between two brain regions (ROIs), reflecting the strength of their FC and corresponding PET change covariance. For each network, the number of ROI pairs per network ranging from 28 to 703, depending on the number of ROIs in each network (ROI pairs per network: VN: n = 91; SMN: n = 91; AN: n = 28; LN: n = 153; FPN: n = 66; DMN: n = 231; SUB: n = 703; BN: n = 66). All *p*-values were FDR-corrected to account for multiple comparisons. Abbreviations: VN = Visual Network; SMN = Sensorimotor Network; AN = Attention Network; LN = Limbic Network; FPN = Frontoparietal Network; DMN = Default Mode Network; SUB = Subcortical Network; BN = Brainstem Network

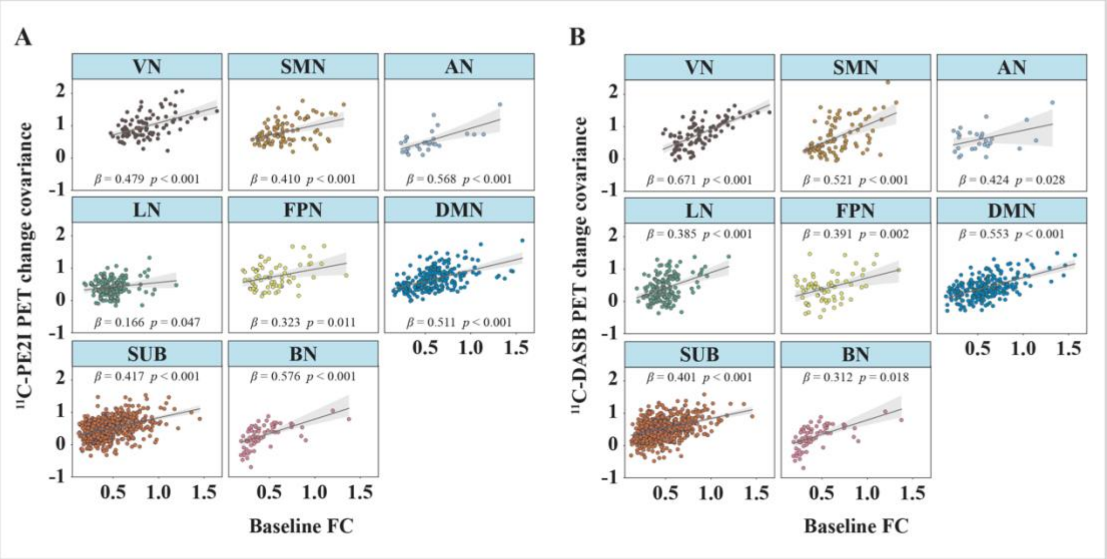

Supplementary Figure 5: **Brain regions in which the association between baseline FC and change in PET uptake is correlated with changes in clinical measures in PD patients.** For each patient (N = 15) and each tracer, FC–PET association maps were generated by performing linear regression at each seed ROI, with the vectorised FC to 137 target ROIs as the independent variable and the corresponding PET uptake change (follow-up – baseline) as the dependent variable. This resulted in a 138-element β-vector per patient representing FC–PET coupling across the brain. Spearman's correlation was then used to identify seed regions where these β-values were significantly associated with changes in clinical symptom severity scores (motor: MDS-UPDRS-III; non-motor: NMSS, BDI, AES, etc.). The figure only shows brain regions with FDR-corrected $p < 0.05$. Abbreviations: UPDRS = Unified Parkinson's disease rating scale; BR sub-score = bradykinesia-rigidity sub-score; BDI = Beck's depression inventory; NMSS = Non-Motor Symptoms Scale; AES = Apathy Evaluation Scale; ACE-R = Addenbrooke's Cognitive Examination Revised; MMSE = Mini-mental State Examination; PDSS = Parkinson's disease sleep scale； VN = Visual Network; SMN = Sensorimotor Network; AN = Attention Network; LN = Limbic Network; FPN = Frontoparietal Network; DMN = Default Mode Network; SUB = Subcortical Network; BN = Brainstem Network； Abbreviations for brain regions shown in the figure are listed in Supplementary Table 1.

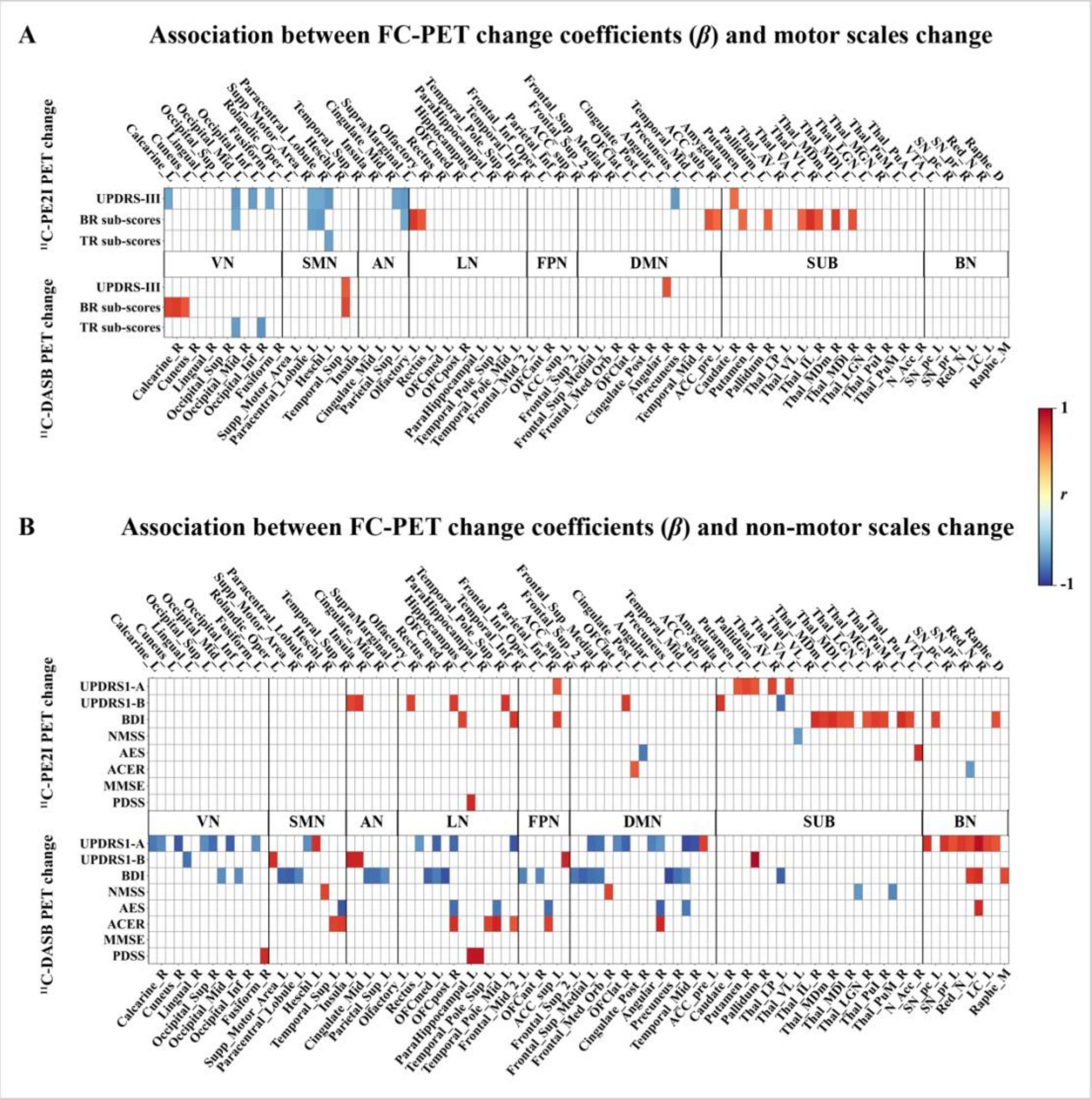
A
Association between FC-PET change coefficients (β) and motor scales change
11C-PE2I PET change
UPDRS-III
BR sub-scores
TR sub-scores
VN
SMN
AN
LN
FPN
DMN
SUB
BN
11C-DASB PET change
UPDRS-III
BR sub-scores
TR sub-scores
1
r
-1
B
Association between FC-PET change coefficients (β) and non-motor scales change
11C-PE2I PET change
UPDRS1-A
UPDRS1-B
BDI
NMSS
AES
ACER
MMSE
PDSS
VN
SMN
AN
LN
FPN
DMN
SUB
BN
11C-DASB PET change
UPDRS1-A
UPDRS1-B
BDI
NMSS
AES
ACER
MMSE
PDSS

Supplementary Figure 6: **Brain regions in which the association between baseline FC and change in PET uptake is correlated with ACER sub-scales (A) and changes in ACER sub-scales in PD patients.** For each patient (N = 30 at baseline and N = 15 at follow-up) and each tracer, FC–PET association maps were generated by performing linear regression at each seed ROI, with the vectorised FC to 137 target ROIs as the independent variable and the corresponding PET uptake change (follow-up – baseline) as the dependent variable. This resulted in a 138-element β-vector per patient representing FC–PET coupling across the brain. Spearman's correlation was then used to identify seed regions where these β-values were significantly associated with ACER sub-scales and changes in ACER sub-scales. The figure only shows brain regions with FDR-corrected $p < 0.05$. Abbreviations: ACER = Addenbrooke's Cognitive Examination Revised; VN = Visual Network; SMN = Sensorimotor Network; AN = Attention Network; LN = Limbic Network; FPN = Frontoparietal Network; DMN = Default Mode Network; SUB = Subcortical Network; BN = Brainstem Network

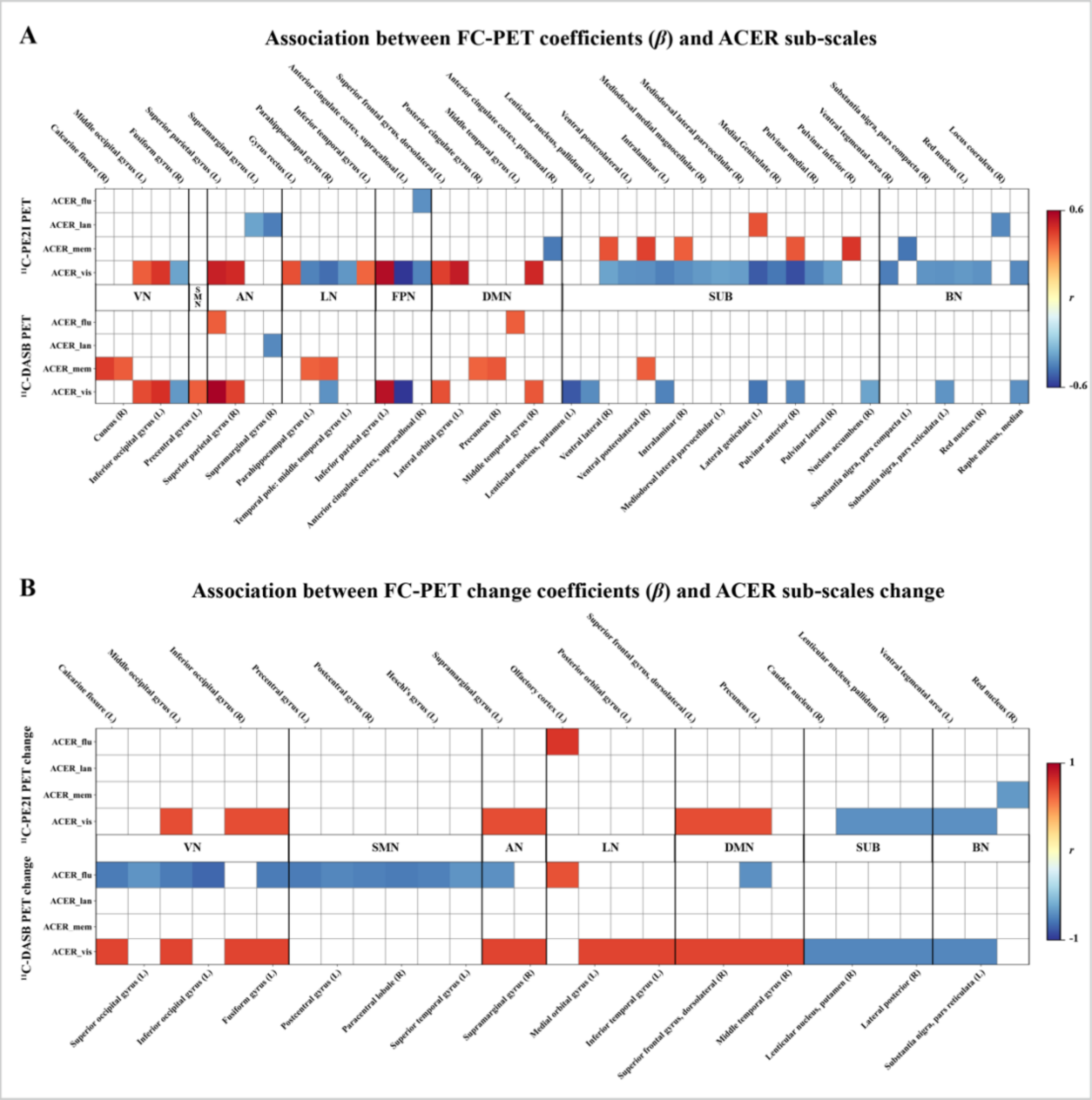

Supplement: fcaf308_Supplementary_Data [file fcaf308_supplementary_data.zip › Supplementary_Materials.pdf]
